# Microglia at center stage: a comprehensive review about the versatile and unique residential macrophages of the central nervous system

**DOI:** 10.18632/oncotarget.23106

**Published:** 2017-12-11

**Authors:** Nils Lannes, Elisabeth Eppler, Samar Etemad, Peter Yotovski, Luis Filgueira

**Affiliations:** ^1^ Albert Gockel, Anatomy, Department of Medicine, University of Fribourg, CH-1700 Fribourg, Switzerland; ^2^ Pestalozzistrasse Zo, Department of BioMedicine, University of Basel, CH-4056 Basel, Switzerland; ^3^ Building 71/218 RBWH Herston, Centre for Clinical Research, The University of Queensland, QLD 4029 Brisbane, Australia

**Keywords:** microglia, neuroinflammation, neurodegeneration, virus infection, brain cancer

## Abstract

Microglia cells are the unique residential macrophages of the central nervous system (CNS). They have a special origin, as they derive from the embryonic yolk sac and enter the developing CNS at a very early stage. They play an important role during CNS development and adult homeostasis. They have a major contribution to adult neurogenesis and neuroinflammation. Thus, they participate in the pathogenesis of neurodegenerative diseases and contribute to aging. They play an important role in sustaining and breaking the blood-brain barrier. As innate immune cells, they contribute substantially to the immune response against infectious agents affecting the CNS. They play also a major role in the growth of tumours of the CNS. Microglia are consequently the key cell population linking the nervous and the immune system. This review covers all different aspects of microglia biology and pathology in a comprehensive way.

## INTRODUCTION

Microglia cells belong to the innate immune system and are the resident tissue macrophages of the central nervous system (CNS), including the brain, the spinal cord, the retina and the olfactory bulb. The term “microglia” was coined by Del Rio-Hortega in the first half of the 20th century [1]. More recently, the embryonic yolk sac macrophages have been identified as microglia precursors that migrate into the CNS [2]. Microglia have a multitude of functions, including support of CNS development and synaptogenesis, sustaining homeostasis and structure, contribution to an immune response against infectious agents and participation in adult neurogenesis, neuroinflammation, degenerative diseases, stroke, trauma and regeneration [3]. Resting ramified microglia build a dense steady-state network of dynamic and reactive cells throughout the nervous tissue that they monitor, scan and control [4]. They interact with all different cell types of the CNS, including neurons [5, 6] and oligodendrocytes [7]. They are important phagocytes and essential for the development of the CNS, as they eliminate apoptotic neurons, produce growth factors and contribute to function and structural organisation of the nerve tissue [8, 9]. Later in life, microglia play a major role in adult neurogenesis [10] and in remodelling of the CNS, especially by contributing to synapse structure and function [11, 12]. Various danger signals can activate microglia, including intrinsic factors derived from cellular and tissue damage, like stroke [13–15] and traumatic injury [16], as well as extrinsic inflammatory factors like cytokines or microbial products [17, 18]. Thereby, microglia responses may be very diverse with a broad range between the two opposite functions, i.e. pro-inflammatory and pro-regeneration responses [19–26]. Microglia often change shape upon activation and may even become migratory [27]. However, activated microglia are the major players in neuroinflammation with possible induction of substantial damage to CNS function and structure. In addition, microglia play an important role in various CNS diseases, including encephalitis [28], Alzheimer’s [29–31] and Parkinson’s disease [32], multiple sclerosis (MS) [33], amyotrophic lateral sclerosis (ALS) [34] and even in mental conditions [35]. Microglia help to sustain the blood-brain barrier (BBB) and upon activation are often responsible for its functional and structural disruption [36, 37] with consecutive invasion of various immune cell types into the CNS [38–41], including blood monocytes [42], granulocytes [43] and lymphocytes [44]. In addition, chronically activated microglia may also be responsible for age-related cognitive and structural decline of the brain [45]. Microglia play also a major role in the immune response against infectious agents that invade the CNS, including viruses [46, 47], bacteria [48] and parasites [49]. Microglia have also a major contribution to development and growth of primary brain tumours, including glioma and glioblastoma, as well as tumour metastasis [50]. Due to the emerging importance of microglia in health and disease of the CNS, various *in silico*, *in vitro* and *in vivo* research models have been developed [51], which resulted in the generation of plenty of new recent knowledge [52] that is to be translated into various promising therapeutic approaches [53]. This review covers all the issues mentioned above, but focusing on immune, infection and inflammatory perspectives. It includes up-to date review articles for important subtopics that are not covered in detail and original research articles for emerging topics. It also emphasizes gaps of knowledge, raises important research related question and suggests areas where more research work may be required.

### Origin of microglia and their role in brain development, synapsogenesis and adult neurogenesis

Microglia are the tissue macrophages of the CNS and serve initially its development and subsequently its homeostasis [54, 55]. The origin of microglia has only recently been discovered [2, 56]. Specialized precursor yolk sac macrophages [57–59] migrate at an early embryonic stage into the developing CNS within a limited time frame, once the cardiovascular development has started [60] and before the blood-brain barrier is closed [61]. Expression of transcription factor PU.1 is essential and defines the microglia lineage, which separates them from other tissue macrophages [62, 63]. Most studies have been done in mouse models, where microglia populate the developing CNS at around E8.5 to E9.5, even before astrocytes and oligodendrocytes emerge [64, 65]. More recently, also zebrafish has been used as a model to study microglia in the embryo, where colonisation takes place around 48hpf [66–70]. Much less is known about early microglia colonisation of the CNS in humans [71, 72].

Expression of the colony-stimulating factor 1 receptor (CD115, M-CSFR, c-*fms*) [73] and the corresponding ligands CSF-1 (M-CSF) and/or IL-34 [74] are essential for the maintenance and expansion of microglia [73, 75]. In addition, GM-CSF and its receptor, as well as neurotrophins sustain survival and proliferation of microglia [76–78]. Initially, neuronal cells provide these factors, before later emerging astrocytes also contribute to their production. However, little is known about spatial expression patterns and the regulation of these factors in the CNS.

**Figure 1:**
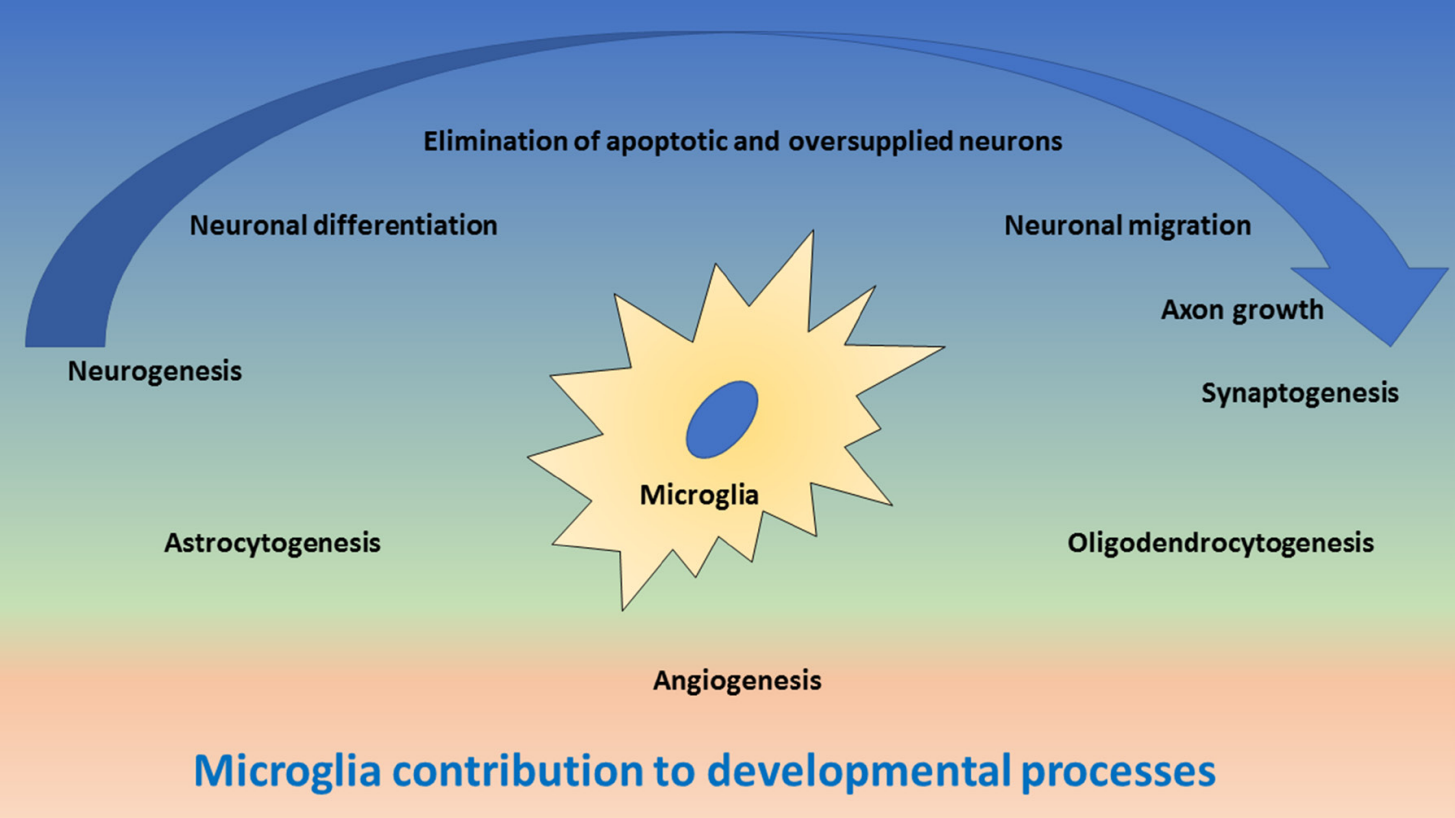
Schematic drawing of important functions of microglia during development of the central nervous system In the upper blue area, the contribution of microglia to neurogenesis and synaptogenesis is depicted, including neuronal differentiation and elimination of apoptotic neurons, neuronal migration and axonal growth. The blue arrow indicates the direction of neuronal development. In the middle green area, the contribution of microglia to astrocytogenesis and oligodendrocytogenesis is depicted. In the lower red area, the contribution of microglia to angiogenesis is depicted.

Microglia have multiple functions in the developing CNS [8], as they contribute to (1) elimination of apoptotic cells and preventing oversupply of neurons, (2) support of neurogenesis, migration and differentiation of neurons, (3) axon growth and synaptogenesis, (4) generation and maturation of astrocytes and oligodendrocytes and (5) angiogenesis (Figure 1). The main mechanisms for these functions are phagocytosis and cell-to-cell communication through direct intercellular contacts or via soluble mediators, which often are still not well understood at cellular and molecular level and which require more future research.

Microglia are attracted to and accumulate in locations of cell death where they engulf apoptotic cells during neurogenesis and migration, differentiation and positioning of the newly generated neurons [66, 79, 80]. Microglia phagocytosis function will be covered in more detail further down. During the early phase of development, the density of microglia is rather low, which is compensated by their increased mobility [81]. Depending on timing and developmental processes, there is a corresponding dynamic spatial patterning of microglia [82–84]. First, they accumulate around areas with proliferation of neuronal precursor cells. Then they line up along the developing axons in the white mater. Later, when neurons have formed the relevant functional structures and have built their wiring connections, supported by astroglia and oligodendroglia, microglia density increases, migration decreases and the cells become highly ramified and remain in their preferred spatial area, whereby as described for the mouse, they are not uniformly shaped across the brain regions [85]. There is a steady-state condition with dying microglia being replaced through proliferation of remaining cells [86]. Of note, the areas covered by ramified microglia do not overlap, in comparison with the area covered by astrocytes [87]. Only little is known about the factors and molecular mechanisms controlling colonisation, migration and settling of the cells, although α5β1 integrin and fibronectins, as well as γ-secretase, seem to play an important role [88, 81]. In addition, little is known about the molecular mechanisms of the interaction between microglia and neurons. However, CX3CL1 (fractalkine) that is expressed on neurons and its receptor CX3CR1 that is expressed on microglia mediate the intercellular communication [5, 89, 90]. Of note, absence or functional deficiency of microglia or the interaction between microglia and neurons result in oversupply of newly generated neurons and accumulation of apoptotic cells in the developing brain leading to functional and structural brain deficiencies [9, 91–96].

As soon as neurons have found their appropriate location, they grow their dendrites and axon, and synaptogenesis emerges, in which microglia play an important role [9, 11, 12, 97–99]. Microglia are essential for the pruning of synapsis, synaptic maturation and the subsequent synaptic communication [4]. In the case of glutamatergic excitatory neurons, pre- and post-synaptic structures are supported and controlled by the interaction between the neurons, astrocytes and the microglia forming a quad-partite synapse [100, 101]. Interestingly, complement factors C1 and C3, as well as the corresponding receptor CR3 (also termed CD11b), which is a β2-integrin, play an important role in microglia mediated synapse modification [102], as such that astrocyte-mediated activation of C1q, the initiator of the classical complement cascade, has been suggested to result in downstream activation of C3b, which then deposits on neurites, thus “tagging” synapse for elimination [103].

Microglia link the immune system with the nerve system, but also respond to endocrine events. In that respect, immune-related or endocrine pathologies may affect microglia functions during development, leading to subsequent functional or even mental disturbance [104, 105]. For mammals, these disturbances can happen during the foetal phase in the course of maternal inflammation or infections, or subsequently because of neonatal systemic immune activation and inflammation [106–109] or stressful social events [110, 111]. Interestingly, it has recently also been proposed that a pathological gut microbiome may somehow act on microglia and their function, resulting in certain mental diseases [112, 113]. However, more neuroimmunology research is needed to get better understanding of long-term influence of microglia deficiencies and pathologies during development on post-natal and adult mental health.

After the embryonic stage, there is a natural turnover of bone marrow derived monocytes that find their way through the blood circulation into the meninges, the perivascular space of the CNS and across the choroid plexus into the cerebral liquor space, from where these monocytes may be quickly recruited into the CNS if required [114–118]. CCR2 expressing monocytes have thereby been identified as a key population that immigrate into the CNS [119], integrate within the microglia network and may no longer be separated from the resident microglia population.

Microglia also contributes substantially to adult neurogenesis [10, 120–123], which takes place in the dentate gyrus for the hippocampus [124], the subventricular zone for the olfactory system [125, 126] and in the hypothalamus for the neuro-endocrine system [127]. Life-long adult neurogenesis is required for the function of memory, olfaction, neuroendocrine system and possibly others. As for the development of the CNS, in adult neurogenesis, enhanced or reduced generation of new neurons and their integration may substantially influence the brain function [124]. Neurogenesis is influenced by microglia [128] either by producing corresponding supporting or suppressing factors, or by eliminating new neurons [129], as well as by sustaining new neurons alive. Thereby, microglia may be influenced in their function by local [130] and systemic factors [131], including fractalkine (CX3CL1) [132], TLR9 [133] and Wnt signalling [134], LPS [135, 136], progranulin [137], allergic reactions [138], systemic inflammation [139], vaccines [140] and vaccination [141], dietary factors [142], exercise [143] and aging [144]. Recently, there are also first experimental attempts to control the influence of microglia on adult neurogenesis via drug therapies, including with indomethacin [145] and minocycline [146]. However, more research is required to better understand the cellular and molecular mechanism of how microglia and systemic immune events influence adult neurogenesis.

### Phenotype and function of microglia

**Figure 2:**
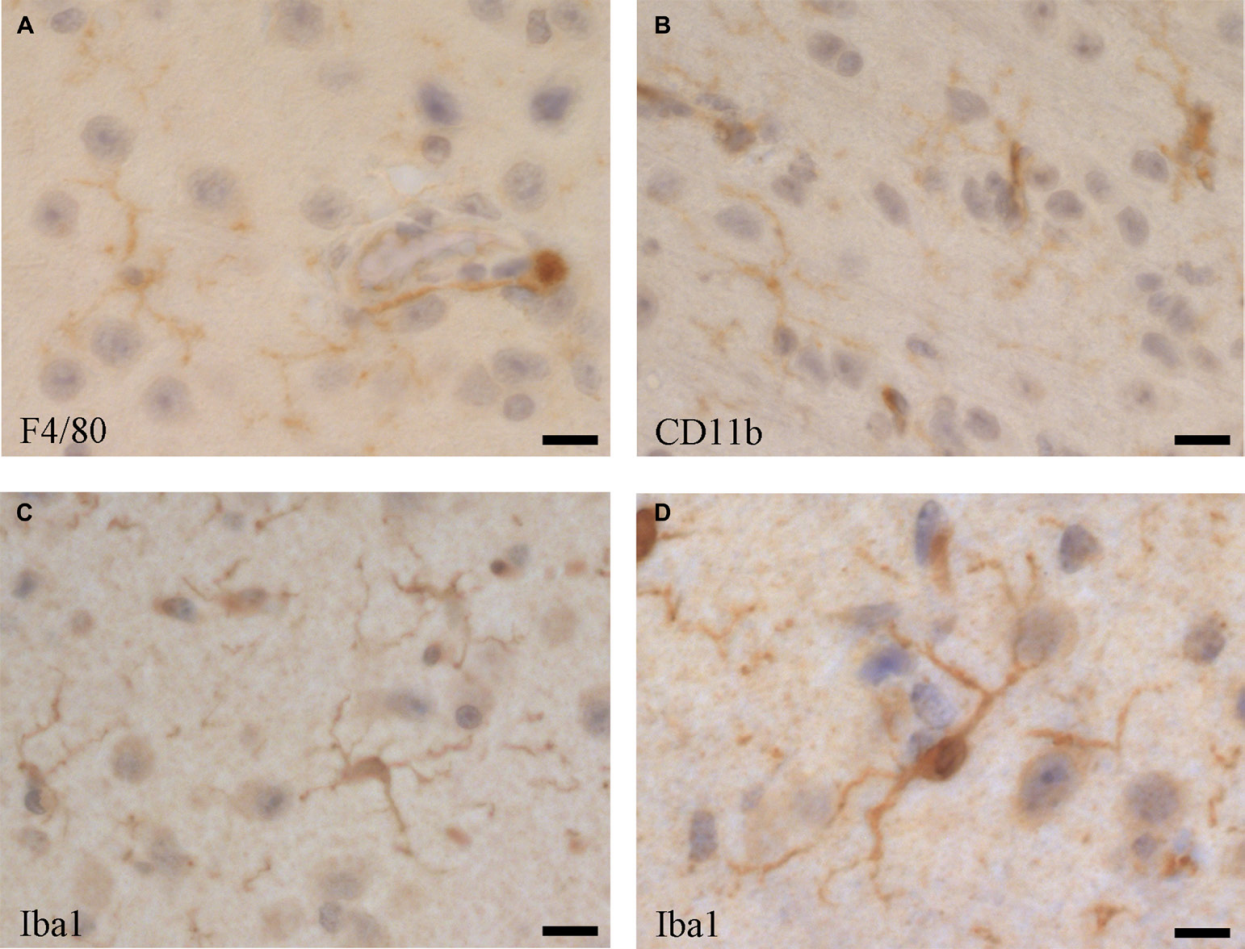
Microglia cells visualised by immune histochemistry in the mouse brain using antibodies directed against (**A**) F4/80 (Serotec, Kidlington, UK, clone Cl:A3-1), (**B**) CD11b (BD Pharmingen, San Diego, USA, cat. no. 553308), and (**C** and **D**) Iba1 (Abcam, UK, cat. no. ab5076). Bars: (A and C) 25 µm, (B) 20 µm, (D) 40 µm.

Upon their discovery, microglia have been identified and differentiated from other CNS-associated myeloid cell populations morphologically through histology. Thereby complex shape and distribution patterns have been described, depending on animal species, location in the CNS and activation stage [87]. Despite their different origin by early immigration from the yolk sac, microglia share many markers with other macrophage populations, such as the blood-derived perivascular, choroid plexus and leptomeningeal macrophages, including F4/80 (Figure 2A), CD11b (Figure 2B) [118], but as microglia usually represent the majority of immune cells in the CNS, some of those markers are routinely used for their characterization [147–149]. Iba1 (ionized calcium binding adaptor molecule 1), which is highly conserved in mammals, has been useful as a specific marker for the detection of microglia, since its discovery [150, 151], as it is not expressed in blood monocytes, but often also in blood-derived tissue macrophages and dendritic cells [118, 152, 153]. Thus, to distinguish microglia from blood-derived immigrated macrophage populations, and from blood monocytes, reduced expression of the common leukocyte antigen CD45 as a marker has been suggested, although CD45 is upregulated in activated microglia [152]. More recently, a plenitude of membrane proteins has been identified in microglia. CX3CR1 (fractalkine receptor) is one important functional membrane protein, as its ligand (fractalkine, CX3CL1) is expressed on neurons [5, 90] and astrocytes [154]. CX3CR1 has been widely used as a marker for flow cytometry and immune histochemistry, as well as for developing a crucial CX3CR1-GFP mouse model, which is functionally very close to the Iba1-GFP model [155]. However, when it comes to identifying newly immigrated monocytic cells into the CNS and to differentiate them from resident microglia, reliable markers are still missing.

The transcriptome analysis of mouse microglia has revealed a unique signature for the freshly isolated brain-derived cells, whereas the cultured cells display properties of activated microglia [156]. In addition to some transcription factors (Rhox5, Cebpe, E2f6, Hoxc6, Phf17, Ppargc1b), several membrane proteins have been identified in microglia that are unique and not expressed in other macrophages, including the ion transporters Slco4a1, Slc30a5, Mcoln3, the lipid-metabolism associated cell membrane molecules Lrp8, Lpcat3, Stab1, Pap2c, and the putative efflux cell membrane receptor Mfsd10. Unfortunately, reliable antibodies against these proteins, for specific histological detection of microglia in the CNS, are still missing. Similar work has also been done with human brain-derived microglia, which shows substantial similarities to the mouse microglia under physiological resting conditions [157]. However, activated microglia and cells from aged individuals show significant differences between mouse and human, as well as depending on location and condition [158, 159], making comparison of transcriptome studies quite difficult [160].

Microglia build a 3-dimensional network in the CNS and they communicate also through hemichannels and gap junctions [161, 162]. The hemichannels allow secretion and uptake of glutamate and ATP, factors that are essential in the communication with neurons and astrocytes. The gap junctions allow microglia to react as a syncytium. However, the extent of such connections and their relevance need further investigation, also in respect of possible pharmacological treatment by using functional modulators or blockers.

Microglia are the professional phagocytes of the CNS. They are able to sense and take up extracellular material, like cell debris, apoptotic cells, as well as tumour cells and microbes. Consequently, they contribute substantially to the function and structure of the CNS. They express a variety of sensing and binding receptors on their surface membrane [163]. Phagocytosis is essential for the control of newly generated neurons during development and adult neurogenesis, as well as for synapse homeostasis. However, they are able to engulf whole or parts of neurons, which may become fatal if phagocytosis gets out of control and if live, functional neurons are eliminated [164–166]. Microglia use a variety of receptors for the recognition of molecules, particles and cells that they engulf [167]. Sialic acid binding immunoglobulin-like lectins (Siglecs) are important regulatory receptors expressed on microglia and binding to sialated ligands on neurons or CNS tumor cells [168–171]. Siglecs signaling modulates activation of microglia and thus also phagocytosis activity. Although they also serve as binding receptors, signaling through SIRPα (signal-regulatory protein alpha; CD172a), complement receptor 3 (CR3; CD11b), LRP (low-density lipoprotein receptor-related protein; CD90.2) and TREM2 (protein triggering receptor expressed on myeloid cells-2) also modulates phagocytosis by microglia, indicating that live neurons can control phagocytosis through expression of corresponding ligands [165, 172–179]. On the other hand, activated microglia secret inflammatory cytokines or other mediators that are able to regulate expression of those ligands on neurons, which may eliminate live and functional neurons and induce disease. Consequently, fine-tuning of the balance between eat-me and don’t-eat-me signals, in the interaction between microglia and neurons, controls for whether neurons are engulfed and eliminated, or not [164]. Interestingly, there seem to be sex and age differences, when it comes to microglia functions, including phagocytosis, although the mechanisms are not well understood [180, 181]. For instance, in experimental autoimmune encephalomyelitis (EAE) mouse model for MS, which may present with reduced relapses during pregnancy, estrogen was found to promote anti-inflammatory, protective and regenerative microglia [182, 183]. Further, in experimental stroke, smaller infarcts and anti-inflammatory microglia were observed in female, but not in male mice [184]. Thus, phagocytosis by microglia is crucial for function and structure of the CNS, as well as in many pathologies. Therefore, more research on this topic is required in the future.

Continuous and sufficient blood supply to the CNS is essential and disruption of perfusion results in subsequent damage of function and structure [185, 186]. Regional perfusion of the CNS is tightly regulated at capillary level depending on oxygen and energy needs by the local neurons, which control regional perfusion indirectly via astrocytes. However, the molecular mechanisms for the regional perfusion control are still not well understood. Most important is also the tight separation of the CNS tissue and intercellular space from the intravascular space, which is given by the blood-brain-barrier (BBB) [187, 188]. The BBB is important to keep serum proteins (e.g. complement system, antibodies, etc.) and a plenitude of soluble factors (e.g. cytokines, microbial products, etc.) in the blood circulation out of the CNS tissue, as many of those factors induce immediate and strong activation of microglia that results in devastating neuroinflammation [189, 190]. In mouse, the CNS vascularisation starts at about E8 by endothelial cells forming a capillary network, at around the same time of microglia colonisation, but before astrocytes emerge. However, CNS pericytes that stabilise the endothelial capillaries emerge around the same time [191]. Yet unknown neuronal factors and the pericytes control differentiation of CNS capillary endothelial cells and thus the formation of the first line of the BBB by increasing the tight connections between endothelial cells and by forcing them to establish limited, CNS-specific transcytosis. The functional capillary system, forming the internal part of the BBB, is composed of a continuous layer of endothelial cells interconnected through tight junctions and enclosing the lumen containing the blood. A distinct continuous basal lamina surrounds the endothelial cells in which pericytes are embedded without forming a continuous layer [192]. The basal lamina and the pericytes form the perivascular space which may also contain immune cells, including macrophages, often referred to as perivascular microglia, and lymphocytes. The perivascular space is peripherally completely covered by endfoot processes of the astrocytes forming the glia limitans, a second, external closure of the BBB interconnected through yet unknown adhesion molecules [193, 194]. Occasionally, also processes of microglia also contribute to the glia limitans layer, called the juxtavascular microglia. Whereas the astrocytes control the transport of nutrients, oxygen and other molecules between the blood vessels and the neurons, the role of the perivascular and juxtavascular microglia is not well understood. However, they may monitor the interface between the first inner and second outer line of the BBB, and consequently mediate between the blood and the CNS environment, and vice-versa. Most important, activated microglia opens up the BBB by releasing inflammatory factors, resulting in enhanced neuroinflammation, by allowing serum components and immune cells entering the CNS tissue [36, 195, 196]. As most recently described [197] in healthy condition, the endothelial BBB is closed and enforced by claudin (CLDN) 5 and . In inflammation, the endothelial part of the BBB downregulates CLDN5 and opens towards the perivascular space, while the astrocytes of the glia limitans upregulate CLDN1, CLDN4, and junctional adhesion molecule A (JAM-A), and forms an enhanced second barrier, composed of reactive astrocytes with tight junctions containing CLDN1, CLDN4 and JAM-A subunits. Enhancement of the second barrier has been attributed to local microglia secreting IL-1β, a driver cytokine of lesion pathogenesis in multiple sclerosis and the corresponding EAE model. In addition to inflammatory diseases of the CNS, stroke and trauma of the CNS are extreme cases of the breakdown of the BBB with subsequent substantial activation of microglia [37, 198, 199]. Activated perivascular microglia may also be able to control repair of the damaged BBB [200, 201], as well as to induce or support angiogenesis, which is important in CNS tumours [202, 203] and vascular pathologies of the retina [204–207]. First experimental therapeutic approaches have been suggested [208], but more research is required. Substantial research of this topic has used live animal imaging [209, 210], often the retina model, as the blood vessels of the eye are easily monitored. However, more *in vivo* research about the influence of microglia on the BBB would be needed and new models ought to be developed.

### Activation of microglia and their role in neuroinflammation, neurodegenerative conditions, mental diseases, aging and gender

**Figure 3:**
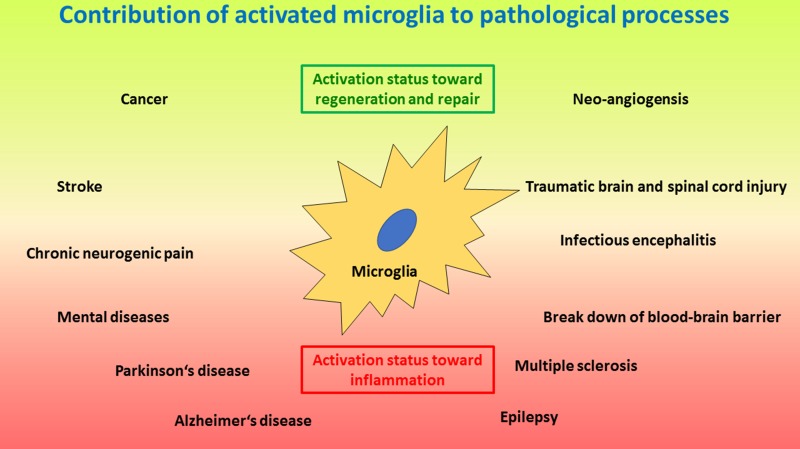
Schematic drawing of important processes where activated microglia play a crucial role The lower red area depicts pathologies induced and/or sustained by microglia activated along the inflammatory and neurotoxic pathway, including neurodegenerative and autoimmune-like diseases, as wells as neurogenic pain, infectious encephalitis and break-down of the blood-brain barrier. The upper green area depicts microglia activated along the regenerative pathway where the focus is on tissue repair, including injury and repair, as well as tumour growth and angioneogenesis. Of note, microglia activation status can be different at different locations at the same time. In addition, microglia activation status may change from one polarized status to the other polarized status over time for certain pathologies; e.g. in infections at first the inflammatory status is required to eliminate the infectious agent, whereas later a regenerative status will support repair of damage caused by the infection.

Under physiological conditions, ramified, resting microglia provides a neuroprotective environment [211, 212]. However, most CNS pathologies, as well as regenerative efforts, include activation of microglia with corresponding inflammatory events (Figure 3) [213, 214]. Activated, inflammatory microglia are thus neurotoxic and kill neurons by engulfing them or releasing various neurotoxic molecules and factors, including reactive oxygen species (ROS), glutamate, Fas-ligand, tumour necrosis factor α (TNFα) and others [215–218]. On the other hand, activated neuroprotective microglia may secrete neurotrophins that support neuroregeneration [219]. Of note, microglia do not act on their own, but they coordinate their action with astroglia [27, 220]. Recently, a new type of microglia has been described under pathological conditions named the ¨dark microglia¨, due to their characteristic dark appearance in electron microscopy because of ultrastructural changes, which are proposed to reflect oxidative stress in a particularly hyperactive subset of microglia [221]. Morphologically, microglia activation results in increased dynamics of the cell processes that are extended and retracted according to corresponding signals. In addition, the cells may give up their home location and migrate towards the area of action close by, where they may accumulate and may form a protective enclosure around the pathological or damaged area usually seen around damaged CNS tissue after trauma or amyloid-β plaques in Alzheimer’s disease [222, 223]. Similar to other tissue macrophages, microglia change upon activation the pattern of surface proteins and secrete various soluble factors [224]. In addition, phagocytosis is upregulated upon activation. Depending on continuous presence or absence of the cause for the activation, or on clearance of the problem, microglia may either display the inflammatory properties and remain chronically activated, or they may change towards a protective phenotype and function where they support tissue repair and restoration of structure and function of the CNS [20, 225, 226]. Interestingly, severe local or systemic events, external to the CNS, like systemic inflammation or bacterial infection with sepsis can open and cross the BBB and subsequently activate microglia [18, 227] and interfere with ongoing CNS processes [228]. On the other hand, activation of microglia and neuroinflammation may also result in the break-down of the BBB and corresponding leakage of complement into the CNS, which results in enhanced activation of microglia and neuroinflammation [229]. However, chronic neuroinflammation may result in neurodegenerative diseases.

Microglia express various pattern recognition receptors (PPRs) for sensing endogenous danger-associated molecular patterns (DAMPs: e.g. heat shock proteins) [230] and exogenous pathogen-associated molecular patterns (PAMPs: microbial proteins, saccharides, lipids, RNA and DNA) [231]. Toll-like receptors (TLRs) are such PPRs, transmit danger signals and strongly activate microglia in the context of CNS damage or infection [232, 233]. Interestingly, galectin-3 (Gal3) secreted by activated microglia is also a ligand of TLR4, which may result in chronic activation of microglia [234]. Amyloid-β, which is found in the context of Alzheimer’s disease, as well as prions also activate microglia [235]. It has also been shown that chromogranin A, released by stressed neurons and recognized by scavenger receptors on microglia results in their activation [217]. Inflammatory activation signals in microglia are then integrated in the inflammasome [236], which results in activation of transcription factors for the transcription of inflammatory genes [237]. Microglia activation can be such a deleterious and destructive event by harming structure and function of the CNS that many regulatory mechanisms ought to be built in this process, of which few have recently been discovered, including TREM2 [238, 239]. Interestingly, upregulation of macrophage colony-stimulating factor receptor (CD115, M-CSFR; c-*fms*) on microglia makes them rather neuroprotective [240], as well as signalling through P2X7 receptor [241]. Like for most immune cells, microglia respond to metabolic and energy-related events and activation results in metabolic reprogramming of the cells [242], which interestingly differs between different activation stages [23], which allows accordingly more detailed differentiation between microglia activation stages. In addition, chronic activated microglia may have substantial epigenetic changes of their chromatin, which may then be difficult to reverse [243].

Trauma of the CNS is a frequent pathology, including traumatic brain (TBI) and spinal cord (SCI) injury. Severity of the damage can range from very minute, e.g. in contusion, to substantial tissue damage resulting in deleterious loss of function, including paresis, paralysis and hemi- or tetraplegia. Microglia are immediately activated in such an event [26] and they try at first to enclose the damage and minimise the spread of it [223]. However, strong activation of microglia may damage viable neighbouring neurons [244]. In the course of the response to injury, microglia contribute first to cleaning debris [245] and later to tissue repair, usually resulting in non-functional scar tissue [21, 27]. Interestingly, minor recurrent trauma like concussion during sport (boxing, soccer, etc.), or injury in a distal CNS region, may also activate microglia in otherwise healthy tissue and result on a longer term in substantial mental disease or chronic pain [246, 247]. One has also to consider that injury breaks down the BBB and allows immigration of blood derived immune cells, including macrophages and lymphocytes that contribute to the acute inflammatory reaction and subsequent tissue repair [16, 248]. Various factors known to contribute to induction and regulation of neuroinflammation, as well as to tissue repair are involved in the course of CNS injury, including CX3CR1-CX3CL1 that mediate communication between microglia and neurons or astrocytes [249]. However, the detailed molecular mechanisms and the extent of the role of microglia in CNS trauma and repair are still not well understood and need further future research [250].

CNS ischemia and stroke are events with similar tissue responses seen in traumatic injury of the CNS, although the cause is different. Correspondingly, microglia reacts very similarly to tissue damage and similarly supports tissue repair mechanisms [13–15, 251–254], also including immigration of peripheral blood leucocytes [255, 256]. Most important is of course to consider the underlying systemic metabolic and inflammatory condition, usually contributing to the cardiovascular disease and subsequent stroke, which may also influence microglia response to ischemia and tissue damage and which increases the complexity of the pathology of stroke [257, 258].

Activated microglia driving chronic neuroinflammation have also been shown to substantially contribute to aging of the CNS [259, 260], epilepsy [261], chronic neuropathic pain [262], mental diseases [35, 263, 264] and neurodegenerative diseases, including Alzheimer’s disease [222], Parkinson’s disease [265], amyotrophic lateral sclerosis (ALS) [34] and multiple sclerosis [33]. Aging goes in parallel with systemic chronic activation of the immune system and polarization towards a low-level inflammatory status [266, 267]. This process also affects the CNS and thus microglia [259, 268], which interferes with CNS homeostasis, especially adult neurogenesis [269], the function and structure of myelination [270] and synapses, as well as the BBB. Mental and neurodegenerative diseases have then probably to be seen as focal processes of age-related activated and dysfunctional microglia [45, 271–274]). However, better understanding of the molecular and cellular mechanisms in the activation of microglia and the regulation of inflammatory processes in the CNS is required and more research is therefore needed in these areas of neuroimmunology.

### The role of microglia in viral encephalitis and in brain tumours

Virus infections are the most frequent reason for encephalitis. A variety of viruses affect the CNS and induce encephalitis, including flaviviruses such as Dengue [275, 276], hepatitis C [277], Japanese encephalitis [278, 279], West Nile [44] and Zika [280–282], as well as herpes viruses such as herpes simplex [283], varicella Zoster [284] and Kaposi’s sarcoma associated herpes virus [285], as well as HIV [286, 287]. All the viruses mentioned above may infect microglia [276–278, 280, 281, 288], whereas flaviviruses and herpes viruses, but not HIV [289], may also infect neurons and/or astroglia. Infected neurons and astroglia usually respond with inflammatory signals that activate surrounding microglia. Infected microglia are usually also activated and may substantially contribute to the anti-viral immune response in the CNS, which results in the recruitment of blood monocytes and T-lymphocytes and in the clearance of the virus. Virus-dependent neuroinflammation often results in the local break-down of the BBB. However, damage of the BBB may not be the initial event for the transmission of the virus from the blood to the CNS tissue and cells, but other mechanisms can be involved. E.g. in the case of Japanese encephalitis, somehow astrocytes and microglia are first infected, before the BBB opens up [290]. Thus, the virus could either get into the CNS via infected capillary endothelial cells which release infectious viral particles towards the CNS, or via infected leucocytes (in HIV) [291] that enter into the CNS and transmit the virus to susceptible microglia [292]. There is also a broad variety of reactivity between different individuals in humans, resulting in a broad spectrum between low and severe neuroinflammation and neuronal infection and cell death. Correspondingly, the infection outcome may vary between asymptomatic and fatal consequences, with a broad spectrum between minor mental disorder and severe cognitive deficiencies. Viral infection of the developing CNS results usually in severe malformation which may even result in a non-viable foetus and thus lead to abortion, as it has been recently documented for Zika [282, 293]. Viral infections of microglia may also interfere with their function, which may result in enhanced, uncontrolled inflammation and/or immune deficiency, of which the mechanisms are still not well understood. Fortunately, vaccines have been successfully established for some viruses, e.g. Japanese encephalitis, which prevents infection in the first instance. However, for many of the mentioned viruses, there is still no vaccine available and substantial more research is required to better understand the role of microglia in corresponding viral encephalitis.

Glioma, especially glioblastoma, is the most frequent primary brain tumour [50]. Therefore, glioma is covered here, representing the principal mechanisms of microglia in CNS primary tumours and metastases. Gliomas develop from genetically aberrant glia cell precursors and are usually monoclonal [294]. Microglia play a role in establishing a growing glioma tumour, and certainly help glioma to grow together with its vascular supply [295–297]. The vascular bed of gliomas has to be considered different from the functional BBB [298] and allows substantial immigration of CCR2 + blood derived monocytes, as well as regulatory/suppressive lymphocytes and other leucocytes into the tissue, due to CCL2 production of the tumour [299, 300]. Interestingly, there is a more immune-suppressive, tumour growth promoting population of residential microglia, as well as an inflammatory, monocytes-derived macrophage population [202]. Together, in the interaction with the tumour cells, they provide an optimal environment for survival and growth of glioma, by preventing an effective anti-tumour immune response, generating space in the healthy tissue and providing growth factors [301–303]. However, future therapies have probably to target, in addition to the tumour cells, also microglia and macrophages, as well as the vascular cells [304]. In addition to mouse and human *in vitro* models, the zebrafish *in vivo* model has become very helpful to investigate the role and interaction of microglia with glioma [305].

### Microglia research models

Microglia research still relies heavily on *in vivo* [51] and *ex vivo* cellular and tissue models [241], including mouse [32, 70, 209, 306, 307], rat [308] and zebrafish [70, 309], as these cells are unique and very different from other tissue macrophages and bone marrow derived cells. However, for research in humans, two *in vitro* approaches have been successfully envisaged: (1) isolation of primary microglia from surgical specimens of the brain [310] or post-mortem tissue samples [147, 278, 311, 312], or (2) differentiation of microglia-like cells from embryonic stem cells [313], induced pluripotent stem cells (iPS) [314–316], bone marrow stem cells or blood monocytes [317–319]. Although all those *in vitro* human models have their limitations, they have so far been very useful to investigate cellular and molecular mechanisms related to cellular structure and function in the context of inflammation, infection and immune response. Recently, microglia from different models have been better characterized using modern transcriptomic methods [320] and compared, indicating that all cultured cells used for *in vitro* models are probably at an activated stage [147], and only *in vivo* models represent physiological resting microglia. However, it may be possible in the future to also develop better *in vitro* models of resting microglia [321] once the *in situ* tissue conditions and molecular and cellular properties are better known. Interestingly, microglia depletion models [322] that can also be replenished with different kinds of modified cells [241] have more recently been developed. It is thus expected that more new models will be developed in the near future in microglia research, adding new knowledge and new aspects of the very versatile and important CNS macrophages, the microglia.

## CONCLUSIONS

Microglia are thus unique cells of the central nervous system, linking it to the immune system. They participate in the biology and pathology of the CNS from early on through-out development and later in CNS homeostasis. Their origin and many of their functions have only recently been discovered, but much more is still not known. Consequently, microglia will remain for the years to come on the centre stage of research in neuroimmunology.

## Abbreviations

(CNS): Central nervous system
(iPS): induced pluripotent stem cells
mS): multiple sclerosis
(CCL): Chemokine ligand
(CCR): Chemokine receptor
(TNFα): tumour necrosis factor alpha
(CD115, M-CSFR; c-*fms*): macrophage colony-stimulating factor receptor
(ROS): reactive oxygen species
(BBB): blood-brain barrier
(HIV): human immune deficiency virus
(IL): interleukin
(CR3, CD11b): complement receptor 3

## References

[1] del Rio-Hortega P (1939). The microglia. Lancet.

[2] Ginhoux F, Greter M, Leboeuf M, Nandi S, See P, Gokhan S, Mehler MF, Conway SJ, Ng LG, Stanley ER, Samokhvalov IM, Merad M (2010). Fate mapping analysis reveals that adult microglia derive from primitive macrophages. Science.

[3] Casano AM, Peri F (2015). Microglia: multitasking specialists of the brain. Dev Cell.

[4] Salter MW, Beggs S (2014). Sublime microglia: expanding roles for the guardians of the CNS. Cell.

[5] Sheridan GK, Murphy KJ (2013). Neuron-glia crosstalk in health and disease: fractalkine and CX3CR1 take centre stage. Open Biol.

[6] Vecino E, Rodriguez FD, Ruzafa N, Pereiro X, Sharma SC (2016). Glia-neuron interactions in the mammalian retina. Prog Retin Eye Res.

[7] Peferoen L, Vogel D, Breur M, Gerritsen W, Dijkstra C, Amor S (2013). Do stressed oligodendrocytes trigger microglia activation in pre-active MS lesion?. Glia.

[8] Frost JL, Schafer DP (2016). Microglia: Architects of the Developing Nervous System. Trends Cell Biol.

[9] Mosser CA, Baptista S, Arnoux I, Audinat E (2017). Microglia in CNS development: shaping the brain for the future. Prog Neurobiol.

[10] Gemma C, Bachstetter AD (2013). The role of microglia in adult hippocampal neurogenesis. Front Cell Neurosci.

[11] Nisticò R, Salter E, Nicolas C, Feligioni M, Mango D, Bortolotto ZA, Gressens P, Collingridge GL, Peineau S (2017). Synaptoimmunology - roles in health and disease. Mol Brain.

[12] Schafer DP, Rosen AR, Lehrman E, Heller C, Stevens B (2013). Pruning CNS synapses: an active role for glia and the complement cascade. J Neurochem.

[13] Anttila JE, Whitaker KW, Wires ES, Harvey BK, Airavaara M (2017). Role of microglia in ischemic focal stroke and recovery: focus on Toll-like receptors. Prog Neuropsychopharmacol Biol Psychiatry.

[14] Guruswamy R, ElAli A (2017). Complex Roles of Microglial Cells in Ischemic Stroke Pathobiology: New Insights and Future Directions. Int J Mol Sci.

[15] Xiong XY, Liu L, Yang QW (2016). Functions and mechanisms of microglia/macrophages in neuroinflammation and neurogenesis after stroke. Prog Neurobiol.

[16] David S, Greenhalgh AD, Kroner A (2015). Macrophage and microglial plasticity in the injured spinal cord. Neuroscience.

[17] Bieber K, Autenrieth SE (2015). Insights how monocytes and dendritic cells contribute and regulate immune defense against microbial pathogens. Immunobiology.

[18] Hoogland IC, Houbolt C, van Westerloo DJ, van Gool WA, van de Beek D (2015). Systemic inflammation and microglial activation: systematic review of animal experiments. J Neuroinflammation.

[19] Ascoli BM, Géa LP, Colombo R, Barbé-Tuana FM, Kapczinski F, Rosa AR (2016). The role of macrophage polarization on bipolar disorder: identifying new therapeutic targets. Aust N Z J Psychiatry.

[20] Cherry JD, Olschowka JA, O’Banion MK (2014). Neuroinflammation and M2 microglia: the good, the bad, and the inflamed. J Neuroinflammation.

[21] Loane DJ, Kumar A (2016). Microglia in the TBI brain: the good, the bad, and the dysregulated. Exp Neurol.

[22] Nakagawa Y, Chiba K (2015). Diversity and plasticity of microglial cells in psychiatric and neurological disorders. Pharmacol Ther.

[23] Orihuela R, McPherson CA, Harry GJ (2016). Microglial M1/M2 polarization and metabolic states. Br J Pharmacol.

[24] Ransohoff RM (2016). A polarizing question: do M1 and M2 microglia exist?. Nat Neurosci.

[25] Tang Y, Le W (2016). Differential Roles of M1 and M2 Microglia in Neurodegenerative Diseases. Mol Neurobiol.

[26] Xu H, Wang Z, Li J, Wu H, Peng Y, Fan L, Chen J, Gu C, Yan F, Wang L, Chen G (2017). The Polarization States of Microglia in TBI: A New Paradigm for Pharmacological Intervention. Neural Plast.

[27] Gao Z, Zhu Q, Zhang Y, Zhao Y, Cai L, Shields CB, Cai J (2013). Reciprocal modulation between microglia and astrocyte in reactive gliosis following the CNS injury. Mol Neurobiol.

[28] Terry RL, Getts DR, Deffrasnes C, van Vreden C, Campbell IL, King NJ (2012). Inflammatory monocytes and the pathogenesis of viral encephalitis. J Neuroinflammation.

[29] Doens D, Fernández PL (2014). Microglia receptors and their implications in the response to amyloid β for Alzheimer’s disease pathogenesis. J Neuroinflammation.

[30] Leyns CE, Holtzman DM (2017). Glial contributions to neurodegeneration in tauopathies. Mol Neurodegener.

[31] Schlachetzki JC, Hüll M (2009). Microglial activation in Alzheimer’s disease. Curr Alzheimer Res.

[32] Joers V, Tansey MG, Mulas G, Carta AR (2017). Microglial phenotypes in Parkinson’s disease and animal models of the disease. Prog Neurobiol.

[33] Luo C, Jian C, Liao Y, Huang Q, Wu Y, Liu X, Zou D, Wu Y (2017). The role of microglia in multiple sclerosis. Neuropsychiatr Dis Treat.

[34] Brites D, Vaz AR (2014). Microglia centered pathogenesis in ALS: insights in cell interconnectivity. Front Cell Neurosci.

[35] Réus GZ, Fries GR, Stertz L, Badawy M, Passos IC, Barichello T, Kapczinski F, Quevedo J (2015). The role of inflammation and microglial activation in the pathophysiology of psychiatric disorders. Neuroscience.

[36] da Fonseca C, Carolina A, Matias D, Garcia C, Amaral R, Geraldo LH, Freitas C, Souza Lima FR (2014). The impact of microglial activation on blood-brain barrier in brain diseases. Front Cell Neurosci.

[37] Dudvarski Stankovic N, Teodorczyk M, Ploen R, Zipp F, Schmidt MH (2016). Microglia-blood vessel interactions: a double-edged sword in brain pathologies. Acta Neuropathol.

[38] Herz J, Filiano AJ, Smith A, Yogev N, Kipnis J (2017). Myeloid Cells in the Central Nervous System. Immunity.

[39] Lively S, Schlichter LC (2013). The microglial activation state regulates migration and roles of matrix-dissolving enzymes for invasion. J Neuroinflammation.

[40] Lopes Pinheiro MA, Kooij G, Mizee MR, Kamermans A, Enzmann G, Lyck R, Schwaninger M, Engelhardt B, de Vries HE (2016). Immune cell trafficking across the barriers of the central nervous system in multiple sclerosis and stroke. Biochim Biophys Acta.

[41] Mracsko E, Javidi E, Na SY, Kahn A, Liesz A, Veltkamp R (2014). Leukocyte invasion of the brain after experimental intracerebral hemorrhage in mice. Stroke.

[42] Varvel NH, Neher JJ, Bosch A, Wang W, Ransohoff RM, Miller RJ, Dingledine R (2016). Infiltrating monocytes promote brain inflammation and exacerbate neuronal damage after status epilepticus. Proc Natl Acad Sci USA.

[43] Neumann J, Riek-Burchardt M, Herz J, Doeppner TR, König R, Hütten H, Etemire E, Männ L, Klingberg A, Fischer T, Görtler MW, Heinze HJ, Reichardt P (2015). Very-late-antigen-4 (VLA-4)-mediated brain invasion by neutrophils leads to interactions with microglia, increased ischemic injury and impaired behavior in experimental stroke. Acta Neuropathol.

[44] Bréhin AC, Mouriès J, Frenkiel MP, Dadaglio G, Desprès P, Lafon M, Couderc T (2008). Dynamics of immune cell recruitment during West Nile encephalitis and identification of a new CD19+B220-BST-2+ leukocyte population. J Immunol.

[45] Spittau B (2017). Aging Microglia-Phenotypes, Functions and Implications for Age-Related Neurodegenerative Diseases. Front Aging Neurosci.

[46] Das Sarma J (2014). Microglia-mediated neuroinflammation is an amplifier of virus-induced neuropathology. J Neurovirol.

[47] Furr SR, Marriott I (2012). Viral CNS infections: role of glial pattern recognition receptors in neuroinflammation. Front Microbiol.

[48] Barichello T, Generoso JS, Simões LR, Goularte JA, Petronilho F, Saigal P, Badawy M, Quevedo J (2016). Role of Microglial Activation in the Pathophysiology of Bacterial Meningitis. Mol Neurobiol.

[49] Morocoima A, Socorro G, Avila R, Hernández A, Merchán S, Ortiz D, Primavera G, Chique J, Herrera L, Urdaneta-Morales S (2012). Trypanosoma cruzi: experimental parasitism in the central nervous system of albino mice. Parasitol Res.

[50] Poon CC, Sarkar S, Yong VW, Kelly JJ (2017). Glioblastoma-associated microglia and macrophages: targets for therapies to improve prognosis. Brain.

[51] Sieger D, Peri F (2013). Animal models for studying microglia: the first, the popular, and the new. Glia.

[52] Atallah N, Vasiu R, Boşca AB, Creţu DI, Georgiu C, Constantin AM, Sovrea AS (2014). Microglia—performers of the 21st century. Rom J Morphol Embryol.

[53] Peña-Altamira E, Prati F, Massenzio F, Virgili M, Contestabile A, Bolognesi ML, Monti B (2016). Changing paradigm to target microglia in neurodegenerative diseases: from anti-inflammatory strategy to active immunomodulation. Expert Opin Ther Targets.

[54] Masuda T, Prinz M (2016). Microglia: A Unique Versatile Cell in the Central Nervous System. ACS Chem Neurosci.

[55] Tay TL, Savage JC, Hui CW, Bisht K, Tremblay MÈ (2017). Microglia across the lifespan: from origin to function in brain development, plasticity and cognition. J Physiol.

[56] Kierdorf K, Erny D, Goldmann T, Sander V, Schulz C, Perdiguero EG, Wieghofer P, Heinrich A, Riemke P, Hölscher C, Müller DN, Luckow B, Brocker T (2013). Microglia emerge from erythromyeloid precursors via Pu.1- and Irf8-dependent pathways. Nat Neurosci.

[57] Gomez Perdiguero E, Klapproth K, Schulz C, Busch K, Azzoni E, Crozet L, Garner H, Trouillet C, de Bruijn MF, Geissmann F, Rodewald HR (2015). Tissue-resident macrophages originate from yolk-sac-derived erythro-myeloid progenitors. Nature.

[58] Hoeffel G, Ginhoux F (2015). Ontogeny of Tissue-Resident Macrophages. Front Immunol.

[59] Nayak D, Roth TL, McGavern DB (2014). Microglia development and function. Annu Rev Immunol.

[60] Walls JR, Coultas L, Rossant J, Henkelman RM (2008). Three-dimensional analysis of vascular development in the mouse embryo. PLoS One.

[61] Tay TL, Hagemeyer N, Prinz M (2016). The force awakens: insights into the origin and formation of microglia. Curr Opin Neurobiol.

[62] Goldmann T, Wieghofer P, Jordão MJ, Prutek F, Hagemeyer N, Frenzel K, Amann L, Staszewski O, Kierdorf K, Krueger M, Locatelli G, Hochgerner H, Zeiser R (2016). Origin, fate and dynamics of macrophages at central nervous system interfaces. Nat Immunol.

[63] Hoeffel G, Chen J, Lavin Y, Low D, Almeida FF, See P, Beaudin AE, Lum J, Low I, Forsberg EC, Poidinger M, Zolezzi F, Larbi A (2015). C-Myb(+) erythro-myeloid progenitor-derived fetal monocytes give rise to adult tissue-resident macrophages. Immunity.

[64] Minocha S, Valloton D, Arsenijevic Y, Cardinaux JR, Guidi R, Hornung JP, Lebrand C (2017). Nkx2.1 regulates the generation of telencephalic astrocytes during embryonic development. Sci Rep.

[65] Petrik D, Yun S, Latchney SE, Kamrudin S, LeBlanc JA, Bibb JA, Eisch AJ (2013). Early postnatal *in vivo* gliogenesis from nestin-lineage progenitors requires cdk5. PLoS One.

[66] Casano AM, Albert M, Peri F (2016). Developmental Apoptosis Mediates Entry and Positioning of Microglia in the Zebrafish Brain. Cell Reports.

[67] Lyons DA, Talbot WS (2015). Glial Cell Development and Function in Zebrafish. Cold Spring Harb Perspect Biol.

[68] Rossi F, Casano AM, Henke K, Richter K, Peri F (2015). The SLC7A7 Transporter Identifies Microglial Precursors prior to Entry into the Brain. Cell Reports.

[69] Svahn AJ, Graeber MB, Ellett F, Lieschke GJ, Rinkwitz S, Bennett MR, Becker TS (2013). Development of ramified microglia from early macrophages in the zebrafish optic tectum. Dev Neurobiol.

[70] Yu T, Guo W, Tian Y, Xu J, Chen J, Li L, Wen Z (2017). Distinct regulatory networks control the development of macrophages of different origins in zebrafish. Blood.

[71] Semple BD, Blomgren K, Gimlin K, Ferriero DM, Noble-Haeusslein LJ (2013). Brain development in rodents and humans: identifying benchmarks of maturation and vulnerability to injury across species. Prog Neurobiol.

[72] Smith AM, Dragunow M (2014). The human side of microglia. Trends Neurosci.

[73] Elmore MR, Najafi AR, Koike MA, Dagher NN, Spangenberg EE, Rice RA, Kitazawa M, Matusow B, Nguyen H, West BL, Green KN (2014). Colony-stimulating factor 1 receptor signaling is necessary for microglia viability, unmasking a microglia progenitor cell in the adult brain. Neuron.

[74] Solary E, Droin N (2014). The emerging specificities of interleukin-34. J Leukoc Biol.

[75] Greter M, Lelios I, Pelczar P, Hoeffel G, Price J, Leboeuf M, Kündig TM, Frei K, Ginhoux F, Merad M, Becher B (2012). Stroma-derived interleukin-34 controls the development and maintenance of langerhans cells and the maintenance of microglia. Immunity.

[76] Koguchi K, Nakatsuji Y, Okuno T, Sawada M, Sakoda S (2003). Microglial cell cycle-associated proteins control microglial proliferation *in vivo* and *in vitro* and are regulated by GM-CSF and density-dependent inhibition. J Neurosci Res.

[77] Salimi K, Moser K, Zassler B, Reindl M, Embacher N, Schermer C, Weis C, Marksteiner J, Sawada M, Humpel C (2002). Glial cell line-derived neurotrophic factor enhances survival of GM-CSF dependent rat GMIR1-microglial cells. Neurosci Res.

[78] Zhang J, Geula C, Lu C, Koziel H, Hatcher LM, Roisen FJ (2003). Neurotrophins regulate proliferation and survival of two microglial cell lines *in vitro*. Exp Neurol.

[79] Svahn AJ, Giacomotto J, Graeber MB, Rinkwitz S, Becker TS (2016). miR-124 Contributes to the functional maturity of microglia. Dev Neurobiol.

[80] Xu J, Wang T, Wu Y, Jin W, Wen Z (2016). Microglia Colonization of Developing Zebrafish Midbrain Is Promoted by Apoptotic Neuron and Lysophosphatidylcholine. Dev Cell.

[81] Smolders SM, Swinnen N, Kessels S, Arnauts K, Smolders S, Le Bras B, Rigo JM, Legendre P, Brône B (2017). Age-specific function of α5β1 integrin in microglial migration during early colonization of the developing mouse cortex. Glia.

[82] Riquier AJ, Sollars SI (2017). Microglia density decreases in the rat rostral nucleus of the solitary tract across development and increases in an age-dependent manner following denervation. Neuroscience.

[83] Schafer DP, Stevens B (2015). Microglia Function in Central Nervous System Development and Plasticity. Cold Spring Harb Perspect Biol.

[84] Swinnen N, Smolders S, Avila A, Notelaers K, Paesen R, Ameloot M, Brône B, Legendre P, Rigo JM (2013). Complex invasion pattern of the cerebral cortex bymicroglial cells during development of the mouse embryo. Glia.

[85] Gordon S, Plüddemann A (2017). Tissue macrophages: heterogeneity and functions. BMC Biol.

[86] Askew K, Li K, Olmos-Alonso A, Garcia-Moreno F, Liang Y, Richardson P, Tipton T, Chapman MA, Riecken K, Beccari S, Sierra A, Molnár Z, Cragg MS (2017). Coupled Proliferation and Apoptosis Maintain the Rapid Turnover of Microglia in the Adult Brain. Cell Reports.

[87] Walker FR, Beynon SB, Jones KA, Zhao Z, Kongsui R, Cairns M, Nilsson M (2014). Dynamic structural remodelling of microglia in health and disease: a review of the models, the signals and the mechanisms. Brain Behav Immun.

[88] Kemmerling N, Wunderlich P, Theil S, Linnartz-Gerlach B, Hersch N, Hoffmann B, Heneka MT, de Strooper B, Neumann H, Walter J (2017). Intramembranous processing by γ-secretase regulates reverse signaling of ephrin-B2 in migration of microglia. Glia.

[89] Arnoux I, Audinat E (2015). Fractalkine Signaling and Microglia Functions in the Developing Brain. Neural Plast.

[90] Paolicelli RC, Bisht K, Tremblay ME (2014). Fractalkine regulation of microglial physiology and consequences on the brain and behavior. Front Cell Neurosci.

[91] Bergon A, Belzeaux R, Comte M, Pelletier F, Hervé M, Gardiner EJ, Beveridge NJ, Liu B, Carr V, Scott RJ, Kelly B, Cairns MJ, Kumarasinghe N (2015). CX3CR1 is dysregulated in blood and brain from schizophrenia patients. Schizophr Res.

[92] Derecki NC, Cronk JC, Lu Z, Xu E, Abbott SB, Guyenet PG, Kipnis J (2012). Wild-type microglia arrest pathology in a mouse model of Rett syndrome. Nature.

[93] Hellwig S, Brioschi S, Dieni S, Frings L, Masuch A, Blank T, Biber K (2016). Altered microglia morphology and higher resilience to stress-induced depression-like behavior in CX3CR1-deficient mice. Brain Behav Immun.

[94] Lehmann ML, Cooper HA, Maric D, Herkenham M (2016). Social defeat induces depressive-like states and microglial activation without involvement of peripheral macrophages. J Neuroinflammation.

[95] Schafer DP, Heller CT, Gunner G, Heller M, Gordon C, Hammond T, Wolf Y, Jung S, Stevens B (2016). Microglia contribute to circuit defects in Mecp2 null mice independent of microglia-specific loss of Mecp2 expression. eLife.

[96] VanRyzin JW, Yu SJ, Perez-Pouchoulen M, McCarthy MM (2016). Temporary Depletion of Microglia during the Early Postnatal Period Induces Lasting Sex-Dependent and Sex-Independent Effects on Behavior in Rats. eNeuro.

[97] Squarzoni P, Oller G, Hoeffel G, Pont-Lezica L, Rostaing P, Low D, Bessis A, Ginhoux F, Garel S (2014). Microglia modulate wiring of the embryonic forebrain. Cell Reports.

[98] Squarzoni P, Thion MS, Garel S (2015). Neuronal and microglial regulators of cortical wiring: usual and novel guideposts. Front Neurosci.

[99] Wake H, Moorhouse AJ, Jinno S, Kohsaka S, Nabekura J (2009). Resting microglia directly monitor the functional state of synapses *in vivo* and determine the fate of ischemic terminals. J Neurosci.

[100] Macht VA (2016). Neuro-immune interactions across development: A look at glutamate in the prefrontal cortex. Neurosci Biobehav Rev.

[101] Mayhew J, Beart PM, Walker FR (2015). Astrocyte and microglial control of glutamatergic signalling: a primer on understanding the disruptive role of chronic stress. J Neuroendocrinol.

[102] Zabel MK, Kirsch WM (2013). From development to dysfunction: microglia and the complement cascade in CNS homeostasis. Ageing Res Rev.

[103] Stevens B, Allen NJ, Vazquez LE, Howell GR, Christopherson KS, Nouri N, Micheva KD, Mehalow AK, Huberman AD, Stafford B, Sher A, Litke AM, Lambris JD (2007). The classical complement cascade mediates CNS synapse elimination. Cell.

[104] Arcuri C, Mecca C, Bianchi R, Giambanco I, Donato R (2017). The Pathophysiological Role of Microglia in Dynamic Surveillance, Phagocytosis and Structural Remodeling of the Developing CNS. Front Mol Neurosci.

[105] Buschert J, Sakalem ME, Saffari R, Hohoff C, Rothermundt M, Arolt V, Zhang W, Ambrée O (2016). Prenatal immune activation in mice blocks the effects of environmental enrichment on exploratory behavior and microglia density. Prog Neuropsychopharmacol Biol Psychiatry.

[106] Claypoole LD, Zimmerberg B, Williamson LL (2017). Neonatal lipopolysaccharide treatment alters hippocampal neuroinflammation, microglia morphology and anxiety-like behavior in rats selectively bred for an infantile trait. Brain Behav Immun.

[107] Fernández de Cossío L, Guzmán A, van der Veldt S, Luheshi GN (2017). Prenatal infection leads to ASD-like behavior and altered synaptic pruning in the mouse offspring. Brain Behav Immun.

[108] Lee JH, Espinera AR, Chen D, Choi KE, Caslin AY, Won S, Pecoraro V, Xu GY, Wei L, Yu SP (2016). Neonatal inflammatory pain and systemic inflammatory responses as possible environmental factors in the development of autism spectrum disorder of juvenile rats. J Neuroinflammation.

[109] Mattei D, Ivanov A, Ferrai C, Jordan P, Guneykaya D, Buonfiglioli A, Schaafsma W, Przanowski P, Deuther-Conrad W, Brust P, Hesse S, Patt M, Sabri O (2017). Maternal immune activation results in complex microglial transcriptome signature in the adult offspring that is reversed by minocycline treatment. Transl Psychiatry.

[110] Wang CY, Cheng CW, Wang WH, Chen PS, Tzeng SF (2016). Postnatal Stress Induced by Injection with Valproate Leads to Developing Emotional Disorders Along with Molecular and Cellular Changes in the Hippocampus and Amygdala. Mol Neurobiol.

[111] Wang HT, Huang FL, Hu ZL, Zhang WJ, Qiao XQ, Huang YQ, Dai RP, Li F, Li CQ (2017). Early-Life Social Isolation-Induced Depressive-Like Behavior in Rats Results in Microglial Activation and Neuronal Histone Methylation that Are Mitigated by Minocycline. Neurotox Res.

[112] Erny D, Hrabě de Angelis AL, Jaitin D, Wieghofer P, Staszewski O, David E, Keren-Shaul H, Mahlakoiv T, Jakobshagen K, Buch T, Schwierzeck V, Utermöhlen O, Chun E (2015). Host microbiota constantly control maturation and function of microglia in the CNS. Nat Neurosci.

[113] Sherwin E, Rea K, Dinan TG, Cryan JF (2016). A gut (microbiome) feeling about the brain. Curr Opin Gastroenterol.

[114] Aguzzi A, Barres BA, Bennett ML (2013). Microglia: scapegoat, saboteur, or something else?. Science.

[115] Asheuer M, Pflumio F, Benhamida S, Dubart-Kupperschmitt A, Fouquet F, Imai Y, Aubourg P, Cartier N (2004). Human CD34+ cells differentiate into microglia and express recombinant therapeutic protein. Proc Natl Acad Sci USA.

[116] Barr CM, Manning J, Lewis CA, Rossi FM, Krieger C (2015). Submyeloablative conditioning with busulfan permits bone marrow-derived cell accumulation in a murine model of Alzheimer’s disease. Neurosci Lett.

[117] Jin N, Gao L, Fan X, Xu H (2017). Friend or Foe? Resident Microglia vs Bone Marrow-Derived Microglia and Their Roles in the Retinal Degeneration. Mol Neurobiol.

[118] Prinz M, Priller J, Sisodia SS, Ransohoff RM (2011). Heterogeneity of CNS myeloid cells and their roles in neurodegeneration. Nat Neurosci.

[119] Mildner A, Schmidt H, Nitsche M, Merkler D, Hanisch UK, Mack M, Heikenwalder M, Brück W, Priller J, Prinz M (2007). Microglia in the adult brain arise from Ly-6ChiCCR2+ monocytes only under defined host conditions. Nat Neurosci.

[120] De Lucia C, Rinchon A, Olmos-Alonso A, Riecken K, Fehse B, Boche D, Perry VH, Gomez-Nicola D (2016). Microglia regulate hippocampal neurogenesis during chronic neurodegeneration. Brain Behav Immun.

[121] Ribeiro Xavier AL, Kress BT, Goldman SA, Lacerda de Menezes JR, Nedergaard M (2015). A Distinct Population of Microglia Supports Adult Neurogenesis in the Subventricular Zone. J Neurosci.

[122] Sato K (2015). Effects of Microglia on Neurogenesis. Glia.

[123] Sierra A, Beccari S, Diaz-Aparicio I, Encinas JM, Comeau S, Tremblay ME (2014). Surveillance, phagocytosis, and inflammation: how never-resting microglia influence adult hippocampal neurogenesis. Neural Plast.

[124] Pistikova A, Brozka H, Stuchlik A (2017). Adult neurogenesis in the hippocampus from a perspective of discrimination and generalization: a hypothesis. Physiol Res.

[125] Lim DA, Alvarez-Buylla A (2016). The Adult Ventricular-Subventricular Zone (V-SVZ) and Olfactory Bulb (OB) Neurogenesis. Cold Spring Harb Perspect Biol.

[126] Shigemoto-Mogami Y, Hoshikawa K, Goldman JE, Sekino Y, Sato K (2014). Microglia enhance neurogenesis and oligodendrogenesis in the early postnatal subventricular zone. J Neurosci.

[127] Maggi R, Zasso J, Conti L (2015). Neurodevelopmental origin and adult neurogenesis of the neuroendocrine hypothalamus. Front Cell Neurosci.

[128] Horgusluoglu E, Nudelman K, Nho K, Saykin AJ (2017). Adult neurogenesis and neurodegenerative diseases: A systems biology perspective. Am J of Med Genet Part B-Neuropsychiatric Genetics.

[129] Luo C, Koyama R, Ikegaya Y (2016). Microglia engulf viable newborn cells in the epileptic dentate gyrus. Glia.

[130] Grier BD, Belluscio L, Cheetham CE (2016). Olfactory Sensory Activity Modulates Microglial-Neuronal Interactions during Dopaminergic Cell Loss in the Olfactory Bulb. Front Cell Neurosci.

[131] Valero J, Paris I, Sierra A (2016). Lifestyle Shapes the Dialogue between Environment, Microglia, and Adult Neurogenesis. ACS Chem Neurosci.

[132] Sellner S, Paricio-Montesinos R, Spieß A, Masuch A, Erny D, Harsan LA, Elverfeldt DV, Schwabenland M, Biber K, Staszewski O, Lira S, Jung S, Prinz M, Blank T (2016). Microglial CX3CR1 promotes adult neurogenesis by inhibiting Sirt 1/p65 signaling independent of CX3CL1. Acta Neuropathol Commun.

[133] Matsuda T, Murao N, Katano Y, Juliandi B, Kohyama J, Akira S, Kawai T, Nakashima K (2015). TLR9 signalling in microglia attenuates seizure-induced aberrant neurogenesis in the adult hippocampus. Nat Commun.

[134] Aniol VA, Tishkina AO, Gulyaeva NV (2016). Neurogenesis and neuroinflammation: the role of Wnt proteins. Neurochem J.

[135] Pang Y, Dai X, Roller A, Carter K, Paul I, Bhatt AJ, Lin RC, Fan LW (2016). Early Postnatal Lipopolysaccharide Exposure Leads to Enhanced Neurogenesis and Impaired Communicative Functions in Rats. PLoS One.

[136] Seong KJ, Lee HG, Kook MS, Ko HM, Jung JY, Kim WJ (2016). Epigallocatechin-3-gallate rescues LPS-impaired adult hippocampal neurogenesis through suppressing the TLR4-NF-κB signaling pathway in mice. Korean J Physiol Pharmacol.

[137] Ma Y, Matsuwaki T, Yamanouchi K, Nishihara M (2017). Progranulin Protects Hippocampal Neurogenesis via Suppression of Neuroinflammatory Responses Under Acute Immune Stress. Mol Neurobiol.

[138] Klein B, Mrowetz H, Thalhamer J, Scheiblhofer S, Weiss R, Aigner L (2016). Allergy Enhances Neurogenesis and Modulates Microglial Activation in the Hippocampus. Front Cell Neurosci.

[139] Chesnokova V, Pechnick RN, Wawrowsky K (2016). Chronic peripheral inflammation, hippocampal neurogenesis, and behavior. Brain Behav Immun.

[140] Qi F, Yang J, Xia Y, Yuan Q, Guo K, Zou J, Yao Z (2016). A(H1N1) vaccination recruits T lymphocytes to the choroid plexus for the promotion of hippocampal neurogenesis and working memory in pregnant mice. Brain Behav Immun.

[141] Yang J, Qi F, Gu H, Zou J, Yang Y, Yuan Q, Yao Z (2016). Neonatal BCG vaccination of mice improves neurogenesis and behavior in early life. Brain Res Bull.

[142] De Luca SN, Ziko I, Sominsky L, Nguyen JC, Dinan T, Miller AA, Jenkins TA, Spencer SJ (2016). Early life overfeeding impairs spatial memory performance by reducing microglial sensitivity to learning. J Neuroinflammation.

[143] Biedermann SV, Auer MK, Bindila L, Ende G, Lutz B, Weber-Fahr W, Gass P, Fuss J (2016). Restricted vs. unrestricted wheel running in mice: effects on brain, behavior and endocannabinoids. Horm Behav.

[144] Weissleder C, Fung SJ, Wong MW, Barry G, Double KL, Halliday GM, Webster MJ, Weickert CS (2016). Decline in Proliferation and Immature Neuron Markers in the Human Subependymal Zone during Aging: Relationship to EGF- and FGF-Related Transcripts. Front Aging Neurosci.

[145] Lopes RS, Cardoso MM, Sampaio AO, Barbosa MS, Souza CC, DA Silva MC, Ferreira EM, Freire MA, Lima RR, Gomes-Leal W (2016). Indomethacin treatment reduces microglia activation and increases numbers of neuroblasts in the subventricular zone and ischaemic striatum after focal ischaemia. J Biosci.

[146] Inta D, Lang UE, Borgwardt S, Meyer-Lindenberg A, Gass P (2017). Microglia Activation and Schizophrenia: Lessons From the Effects of Minocycline on Postnatal Neurogenesis, Neuronal Survival and Synaptic Pruning. Schizophr Bull.

[147] Melief J, Sneeboer MA, Litjens M, Ormel PR, Palmen SJ, Huitinga I, Kahn RS, Hol EM, de Witte LD (2016). Characterizing primary human microglia: A comparative study with myeloid subsets and culture models. Glia.

[148] Melief J, Koning N, Schuurman KG, Van De Garde MD, Smolders J, Hoek RM, Van Eijk M, Hamann J, Huitinga I (2012). Phenotyping primary human microglia: tight regulation of LPS responsiveness. Glia.

[149] Sousa C, Biber K, Michelucci A (2017). Cellular and Molecular Characterization of Microglia: A Unique Immune Cell Population. Front Immunol.

[150] Ahmed Z, Shaw G, Sharma VP, Yang C, McGowan E, Dickson DW (2007). Actin-binding proteins coronin-1a and IBA-1 are effective microglial markers for immunohistochemistry. J Histochem Cytochem.

[151] Imai Y, Ibata I, Ito D, Ohsawa K, Kohsaka S (1996). A novel gene iba1 in the major histocompatibility complex class III region encoding an EF hand protein expressed in a monocytic lineage. Biochem Biophys Res Commun.

[152] Greter M, Lelios I, Croxford AL (2015). Microglia Versus Myeloid Cell Nomenclature during Brain Inflammation. Front Immunol.

[153] Wijesundera KK, Izawa T, Tennakoon AH, Murakami H, Golbar HM, Katou-Ichikawa C, Tanaka M, Kuwamura M, Yamate J (2014). M1- and M2-macrophage polarization in rat liver cirrhosis induced by thioacetamide (TAA), focusing on Iba1 and galectin-3. Exp Mol Pathol.

[154] O’Sullivan SA, Gasparini F, Mir AK, Dev KK (2016). Fractalkine shedding is mediated by p38 and the ADAM10 protease under pro-inflammatory conditions in human astrocytes. J Neuroinflammation.

[155] Hirasawa T, Ohsawa K, Imai Y, Ondo Y, Akazawa C, Uchino S, Kohsaka S (2005). Visualization of microglia in living tissues using Iba1-EGFP transgenic mice. J Neurosci Res.

[156] Beutner C, Linnartz-Gerlach B, Schmidt SV, Beyer M, Mallmann MR, Staratschek-Jox A, Schultze JL, Neumann H (2013). Unique transcriptome signature of mouse microglia. Glia.

[157] Gosselin D, Skola D, Coufal NG, Holtman IR, Schlachetzki JC, Sajti E, Jaeger BN, O’Connor C, Fitzpatrick C, Pasillas MP, Pena M, Adair A, Gonda DD (2017). An environment-dependent transcriptional network specifies human microglia identity. Science.

[158] Doorn KJ, Brevé JJ, Drukarch B, Boddeke HW, Huitinga I, Lucassen PJ, van Dam AM (2015). Brain region-specific gene expression profiles in freshly isolated rat microglia. Front Cell Neurosci.

[159] Noristani HN, Gerber YN, Sabourin JC, Le Corre M, Lonjon N, Mestre-Frances N, Hirbec HE, Perrin FE (2017). RNA-Seq Analysis of Microglia Reveals Time-Dependent Activation of Specific Genetic Programs following Spinal Cord Injury. Front Mol Neurosci.

[160] E Hirbec H, Noristani HN, Perrin FE (2017). Microglia Responses in Acute and Chronic Neurological Diseases: What Microglia-Specific Transcriptomic Studies Taught (and did Not Teach) Us. Front Aging Neurosci.

[161] Gajardo-Gómez R, Labra VC, Orellana JA (2016). Connexins and Pannexins: New Insights into Microglial Functions and Dysfunctions. Front Mol Neurosci.

[162] Madry C, Attwell D (2015). Receptors, ion channels, and signaling mechanisms underlying microglial dynamics. J Biol Chem.

[163] Fu R, Shen Q, Xu P, Luo JJ, Tang Y (2014). Phagocytosis of microglia in the central nervous system diseases. Mol Neurobiol.

[164] Brown GC, Neher JJ (2014). Microglial phagocytosis of live neurons. Nat Rev Neurosci.

[165] Fricker M, Oliva-Martín MJ, Brown GC (2012). Primary phagocytosis of viable neurons by microglia activated with LPS or Aβ is dependent on calreticulin/LRP phagocytic signalling. J Neuroinflammation.

[166] Neniskyte U, Fricker M, Brown GC (2016). Amyloid β induces microglia to phagocytose neurons via activation of protein kinase Cs and NADPH oxidase. Int J Biochem Cell Biol.

[167] Sierra A, Abiega O, Shahraz A, Neumann H (2013). Janus-faced microglia: beneficial and detrimental consequences of microglial phagocytosis. Front Cell Neurosci.

[168] Claude J, Linnartz-Gerlach B, Kudin AP, Kunz WS, Neumann H (2013). Microglial CD33-related Siglec-E inhibits neurotoxicity by preventing the phagocytosis-associated oxidative burst. J Neurosci.

[169] Kopatz J, Beutner C, Welle K, Bodea LG, Reinhardt J, Claude J, Linnartz-Gerlach B, Neumann H (2013). Siglec-h on activated microglia for recognition and engulfment of glioma cells. Glia.

[170] Linnartz-Gerlach B, Mathews M, Neumann H (2014). Sensing the neuronal glycocalyx by glial sialic acid binding immunoglobulin-like lectins. Neuroscience.

[171] Linnartz-Gerlach B, Kopatz J, Neumann H (2014). Siglec functions of microglia. Glycobiology.

[172] Gitik M, Liraz-Zaltsman S, Oldenborg PA, Reichert F, Rotshenker S (2011). Myelin down-regulates myelin phagocytosis by microglia and macrophages through interactions between CD47 on myelin and SIRPα (signal regulatory protein-α) on phagocytes. J Neuroinflammation.

[173] Hadas S, Spira M, Hanisch UK, Reichert F, Rotshenker S (2012). Complement receptor-3 negatively regulates the phagocytosis of degenerated myelin through tyrosine kinase Syk and cofilin. J Neuroinflammation.

[174] Kleinberger G, Brendel M, Mracsko E, Wefers B, Groeneweg L, Xiang X, Focke C, Deußing M, Suárez-Calvet M, Mazaheri F, Parhizkar S, Pettkus N, Wurst W (2017). The FTD-like syndrome causing TREM2 T66M mutation impairs microglia function, brain perfusion, and glucose metabolism. EMBO J.

[175] Kober DL, Brett TJ (2017). TREM2-Ligand Interactions in Health and Disease. J Mol Biol.

[176] Mazaheri F, Snaidero N, Kleinberger G, Madore C, Daria A, Werner G, Krasemann S, Capell A, Trümbach D, Wurst W, Brunner B, Bultmann S, Tahirovic S (2017). TREM2 deficiency impairs chemotaxis and microglial responses to neuronal injury. EMBO Rep.

[177] Werneburg S, Buettner FF, Erben L, Mathews M, Neumann H, Mühlenhoff M, Hildebrandt H (2016). Polysialylation and lipopolysaccharide-induced shedding of E-selectin ligand-1 and neuropilin-2 by microglia and THP-1 macrophages. Glia.

[178] Wu R, Li X, Xu P, Huang L, Cheng J, Huang X, Jiang J, Wu LJ, Tang Y (2017). TREM2 protects against cerebral ischemia/reperfusion injury. Mol Brain.

[179] Yang L, Liu CC, Zheng H, Kanekiyo T, Atagi Y, Jia L, Wang D, N’songo A, Can D, Xu H, Chen XF, Bu G (2016). LRP1 modulates the microglial immune response via regulation of JNK and NF-κB signaling pathways. J Neuroinflammation.

[180] Nelson LH, Warden S, Lenz KM (2017). Sex differences in microglial phagocytosis in the neonatal hippocampus. Brain Behav Immun.

[181] Nissen JC (2017). Microglial Function across the Spectrum of Age and Gender. Int J Mol Sci.

[182] Benedek G, Zhang J, Bodhankar S, Nguyen H, Kent G, Jordan K, Manning D, Vandenbark AA, Offner H (2016). Estrogen induces multiple regulatory B cell subtypes and promotes M2 microglia and neuroprotection during experimental autoimmune encephalomyelitis. J Neuroimmunol.

[183] Benedek G, Zhang J, Nguyen H, Kent G, Seifert HA, Davin S, Stauffer P, Vandenbark AA, Karstens L, Asquith M, Offner H (2017). Estrogen protection against EAE modulates the microbiota and mucosal-associated regulatory cells. J Neuroimmunol.

[184] Seifert HA, Benedek G, Liang J, Nguyen H, Kent G, Vandenbark AA, Saugstad JA, Offner H (2017). Sex differences in regulatory cells in experimental stroke. Cell Immunol.

[185] Hattori Y, Enmi J, Kitamura A, Yamamoto Y, Saito S, Takahashi Y, Iguchi S, Tsuji M, Yamahara K, Nagatsuka K, Iida H, Ihara M (2015). A novel mouse model of subcortical infarcts with dementia. J Neurosci.

[186] Wolf G, Lotan A, Lifschytz T, Ben-Ari H, Kreisel Merzel T, Tatarskyy P, Valitzky M, Mernick B, Avidan E, Koroukhov N, Lerer B (2017). Differentially Severe Cognitive Effects of Compromised Cerebral Blood Flow in Aged Mice: Association with Myelin Degradation and Microglia Activation. Front Aging Neurosci.

[187] Blanchette M, Daneman R (2015). Formation and maintenance of the BBB. Mech Dev.

[188] Daneman R, Prat A (2015). The blood-brain barrier. Cold Spring Harb Perspect Biol.

[189] Bardehle S, Rafalski VA, Akassoglou K (2015). Breaking boundaries-coagulation and fibrinolysis at the neurovascular interface. Front Cell Neurosci.

[190] Weinstein JR, Hong S, Kulman JD, Bishop C, Kuniyoshi J, Andersen H, Ransom BR, Hanisch UK, Möller T (2005). Unraveling thrombin’s true microglia-activating potential: markedly disparate profiles of pharmaceutical-grade and commercial-grade thrombin preparations. J Neurochem.

[191] Daneman R, Zhou L, Kebede AA, Barres BA (2010). Pericytes are required for blood-brain barrier integrity during embryogenesis. Nature.

[192] Sakuma R, Kawahara M, Nakano-Doi A, Takahashi A, Tanaka Y, Narita A, Kuwahara-Otani S, Hayakawa T, Yagi H, Matsuyama T, Nakagomi T (2016). Brain pericytes serve as microglia-generating multipotent vascular stem cells following ischemic stroke. J Neuroinflammation.

[193] Mathiisen TM, Lehre KP, Danbolt NC, Ottersen OP (2010). The perivascular astroglial sheath provides a complete covering of the brain microvessels: an electron microscopic 3D reconstruction. Glia.

[194] Miyata M, Mandai K, Maruo T, Sato J, Shiotani H, Kaito A, Itoh Y, Wang S, Fujiwara T, Mizoguchi A, Takai Y, Rikitake Y (2016). Localization of nectin-2δ at perivascular astrocytic endfoot processes and degeneration of astrocytes and neurons in nectin-2 knockout mouse brain. Brain Res.

[195] Giannoni P, Arango-Lievano M, Neves ID, Rousset MC, Baranger K, Rivera S, Jeanneteau F, Claeysen S, Marchi N (2016). Cerebrovascular pathology during the progression of experimental Alzheimer’s disease. Neurobiol Dis.

[196] Rosenberg GA (2017). Extracellular matrix inflammation in vascular cognitive impairment and dementia. Clin Sci (Lond).

[197] Horng S, Therattil A, Moyon S, Gordon A, Kim K, Argaw AT, Hara Y, Mariani JN, Sawai S, Flodby P, Crandall ED, Borok Z, Sofroniew MV (2017). Astrocytic tight junctions control inflammatory CNS lesion pathogenesis. J Clin Invest.

[198] Cai W, Zhang K, Li P, Zhu L, Xu J, Yang B, Hu X, Lu Z, Chen J (2017). Dysfunction of the neurovascular unit in ischemic stroke and neurodegenerative diseases: an aging effect. Ageing Res Rev.

[199] Lake EM, Bazzigaluppi P, Mester J, Thomason LA, Janik R, Brown M, McLaurin J, Carlen PL, Corbett D, Stanisz GJ, Stefanovic B (2017). Neurovascular unit remodelling in the subacute stage of stroke recovery. Neuroimage.

[200] Bowyer JF, Sarkar S, Tranter KM, Hanig JP, Miller DB, O’Callaghan JP (2016). Vascular-directed responses of microglia produced by methamphetamine exposure: indirect evidence that microglia are involved in vascular repair?. J Neuroinflammation.

[201] Lou N, Takano T, Pei Y, Xavier AL, Goldman SA, Nedergaard M (2016). Purinergic receptor P2RY12-dependent microglial closure of the injured blood-brain barrier. Proc Natl Acad Sci USA.

[202] Brandenburg S, Müller A, Turkowski K, Radev YT, Rot S, Schmidt C, Bungert AD, Acker G, Schorr A, Hippe A, Miller K, Heppner FL, Homey B, Vajkoczy P (2016). Resident microglia rather than peripheral macrophages promote vascularization in brain tumors and are source of alternative pro-angiogenic factors. Acta Neuropathol.

[203] Ghoochani A, Yakubov E, Sehm T, Fan Z, Hock S, Buchfelder M, Eyüpoglu IY, Savaskan NE (2016). A versatile *ex vivo* technique for assaying tumor angiogenesis and microglia in the brain. Oncotarget.

[204] Biesecker KR, Srienc AI, Shimoda AM, Agarwal A, Bergles DE, Kofuji P, Newman EA (2016). Glial Cell Calcium Signaling Mediates Capillary Regulation of Blood Flow in the Retina. J Neurosci.

[205] Chinnery HR, McMenamin PG, Dando SJ (2017). Macrophage physiology in the eye. Pflugers Arch.

[206] Dejda A, Mawambo G, Daudelin JF, Miloudi K, Akla N, Patel C, Andriessen EM, Labrecque N, Sennlaub F, Sapieha P (2016). Neuropilin-1-Expressing Microglia Are Associated With Nascent Retinal Vasculature Yet Dispensable for Developmental Angiogenesis. Invest Ophthalmol Vis Sci.

[207] Talia DM, Deliyanti D, Agrotis A, Wilkinson-Berka JL (2016). Inhibition of the Nuclear Receptor RORγ and Interleukin-17A Suppresses Neovascular Retinopathy: Involvement of Immunocompetent Microglia. Arterioscler Thromb Vasc Biol.

[208] Lake EM, Mester J, Thomason LA, Adams C, Bazzigaluppi P, Koletar M, Janik R, Carlen P, McLaurin J, Stanisz GJ, Stefanovic B (2017). Modulation of the peri-infarct neurogliovascular function by delayed COX-1 inhibition. J Magn Reson Imaging.

[209] Brawek B, Garaschuk O (2017). Monitoring *in vivo* function of cortical microglia. Cell Calcium.

[210] Nishimura C, Polesskaya O, Dewhurst S, Silva JN (2016). Quantification of Cerebral Vascular Architecture using Two-photon Microscopy in a Mouse Model of HIV-induced Neuroinflammation. J Vis Exp.

[211] Chen Z, Trapp BD (2016). Microglia and neuroprotection. J Neurochem.

[212] Vinet J, Weering HR, Heinrich A, Kälin RE, Wegner A, Brouwer N, Heppner FL, Rooijen N, Boddeke HW, Biber K (2012). Neuroprotective function for ramified microglia in hippocampal excitotoxicity. J Neuroinflammation.

[213] Lyman M, Lloyd DG, Ji X, Vizcaychipi MP, Ma D (2014). Neuroinflammation: the role and consequences. Neurosci Res.

[214] Ransohoff RM, Schafer D, Vincent A, Blachère NE, Bar-Or A (2015). Neuroinflammation: Ways in Which the Immune System Affects the Brain. Neurotherapeutics.

[215] Biber K, Owens T, Boddeke E (2014). What is microglia neurotoxicity (Not)?. Glia.

[216] Brown GC, Vilalta A (2015). How microglia kill neurons. Brain Res.

[217] Hooper C, Fry VA, Sevastou IG, Pocock JM (2009). Scavenger receptor control of chromogranin A-induced microglial stress and neurotoxic cascades. FEBS Lett.

[218] Li B, Bedard K, Sorce S, Hinz B, Dubois-Dauphin M, Krause KH (2009). NOX4 expression in human microglia leads to constitutive generation of reactive oxygen species and to constitutive IL-6 expression. J Innate Immun.

[219] Hutchinson AJ, Chou CL, Israel DD, Xu W, Regan JW (2009). Activation of EP2 prostanoid receptors in human glial cell lines stimulates the secretion of BDNF. Neurochem Int.

[220] Liddelow SA, Guttenplan KA, Clarke LE, Bennett FC, Bohlen CJ, Schirmer L, Bennett ML, Münch AE, Chung WS, Peterson TC, Wilton DK, Frouin A, Napier BA (2017). Neurotoxic reactive astrocytes are induced by activated microglia. Nature.

[221] Bisht K, Sharma KP, Lecours C, Sánchez MG, El Hajj H, Milior G, Olmos-Alonso A, Gómez-Nicola D, Luheshi G, Vallières L, Branchi I, Maggi L, Limatola C (2016). Dark microglia: A new phenotype predominantly associated with pathological states. Glia.

[222] Heneka MT, Golenbock DT, Latz E (2015). Innate immunity in Alzheimer’s disease. Nat Immunol.

[223] Hines DJ, Hines RM, Mulligan SJ, Macvicar BA (2009). Microglia processes block the spread of damage in the brain and require functional chloride channels. Glia.

[224] Novoselov VV, Sazonova MA, Ivanova EA, Orekhov AN (2015). Study of the activated macrophage transcriptome. Exp Mol Pathol.

[225] Colton CA (2009). Heterogeneity of microglial activation in the innate immune response in the brain. J Neuroimmune Pharmacol.

[226] Parisi C, Napoli G, Pelegrin P, Volonte C (2016). M1 and M2 Functional Imprinting of Primary Microglia: Role of P2X7 Activation and miR-125b. Mediators Inflamm.

[227] Michels M, Sonai B, Dal-Pizzol F (2017). Polarization of microglia and its role in bacterial sepsis. J Neuroimmunol.

[228] Gyoneva S, Davalos D, Biswas D, Swanger SA, Garnier-Amblard E, Loth F, Akassoglou K, Traynelis SF (2014). Systemic inflammation regulates microglial responses to tissue damage *in vivo*. Glia.

[229] Bodea LG, Wang Y, Linnartz-Gerlach B, Kopatz J, Sinkkonen L, Musgrove R, Kaoma T, Muller A, Vallar L, Di Monte DA, Balling R, Neumann H (2014). Neurodegeneration by activation of the microglial complement-phagosome pathway. J Neurosci.

[230] Holtman IR, Bsibsi M, Gerritsen WH, Boddeke HW, Eggen BJ, van der Valk P, Kipp M, van Noort JM, Amor S (2017). Identification of highly connected hub genes in the protective response program of human macrophages and microglia activated by alpha B-crystallin. Glia.

[231] Saijo K, Crotti A, Glass CK (2013). Regulation of microglia activation and deactivation by nuclear receptors. Glia.

[232] Heiman A, Pallottie A, Heary RF, Elkabes S (2014). Toll-like receptors in central nervous system injury and disease: a focus on the spinal cord. Brain Behav Immun.

[233] Lehnardt S (2010). Innate immunity and neuroinflammation in the CNS: the role of microglia in Toll-like receptor-mediated neuronal injury. Glia.

[234] Burguillos MA, Svensson M, Schulte T, Boza-Serrano A, Garcia-Quintanilla A, Kavanagh E, Santiago M, Viceconte N, Oliva-Martin MJ, Osman AM, Salomonsson E, Amar L, Persson A (2015 Mar 4). Microglia-Secreted Galectin-3 Acts as a Toll-like Receptor 4 Ligand and Contributes to Microglial Activation. Cell Rep.

[235] Tu J, Chen B, Yang L, Qi K, Lu J, Zhao D (2015). Amyloid-β Activates Microglia and Regulates Protein Expression in a Manner Similar to Prions. J Mol Neurosci.

[236] Hanamsagar R, Hanke ML, Kielian T (2012). Toll-like receptor (TLR) and inflammasome actions in the central nervous system. Trends Immunol.

[237] Das A, Chai JC, Kim SH, Lee YS, Park KS, Jung KH, Chai YG (2015). Transcriptome sequencing of microglial cells stimulated with TLR3 and TLR4 ligands. BMC Genomics.

[238] Owens R, Grabert K, Davies CL, Alfieri A, Antel JP, Healy LM, McColl BW (2017). Divergent Neuroinflammatory Regulation of Microglial TREM Expression and Involvement of NF-κB. Front Cell Neurosci.

[239] Zhong L, Zhang ZL, Li X, Liao C, Mou P, Wang T, Wang Z, Wang Z, Wei M, Xu H, Bu G, Chen XF (2017). TREM2/DAP12 Complex Regulates Inflammatory Responses in Microglia via the JNK Signaling Pathway. Front Aging Neurosci.

[240] Mitrasinovic OM, Grattan A, Robinson CC, Lapustea NB, Poon C, Ryan H, Phong C, Murphy GM (2005). Microglia overexpressing the macrophage colony-stimulating factor receptor are neuroprotective in a microglial-hippocampal organotypic coculture system. J Neurosci.

[241] Masuch A, Shieh CH, van Rooijen N, van Calker D, Biber K (2016). Mechanism of microglia neuroprotection: involvement of P2X7, TNFα, and valproic acid. Glia.

[242] Gimeno-Bayón J, López-López A, Rodríguez MJ, Mahy N (2014). Glucose pathways adaptation supports acquisition of activated microglia phenotype. J Neurosci Res.

[243] Staszewski O, Prinz M (2014). Glial epigenetics in neuroinflammation and neurodegeneration. Cell Tissue Res.

[244] Yip PK, Carrillo-Jimenez A, King P, Vilalta A, Nomura K, Chau CC, Egerton AM, Liu ZH, Shetty AJ, Tremoleda JL, Davies M, Deierborg T, Priestley JV (2017). Galectin-3 released in response to traumatic brain injury acts as an alarmin orchestrating brain immune response and promoting neurodegeneration. Sci Rep.

[245] Chang CF, Wan J, Li Q, Renfroe SC, Heller NM, Wang J (2017). Alternative activation-skewed microglia/macrophages promote hematoma resolution in experimental intracerebral hemorrhage. Neurobiol Dis.

[246] Muccigrosso MM, Ford J, Benner B, Moussa D, Burnsides C, Fenn AM, Popovich PG, Lifshitz J, Walker FR, Eiferman DS, Godbout JP (2016). Cognitive deficits develop 1month after diffuse brain injury and are exaggerated by microglia-associated reactivity to peripheral immune challenge. Brain Behav Immun.

[247] Sun R, Zhang Z, Lei Y, Liu Y, Lu C, Rong H, Sun Y, Zhang W, Ma Z, Gu X (2016). Hippocampal activation of microglia may underlie the shared neurobiology of comorbid posttraumatic stress disorder and chronic pain. Mol Pain.

[248] Gao W, Li F, Zhou Z, Xu X, Wu Y, Zhou S, Yin D, Sun D, Xiong J, Jiang R, Zhang J (2017). IL-2/Anti-IL-2 Complex Attenuates Inflammation and BBB Disruption in Mice Subjected to Traumatic Brain Injury. Front Neurol.

[249] Poniatowski ŁA, Wojdasiewicz P, Krawczyk M, Szukiewicz D, Gasik R, Kubaszewski Ł, Kurkowska-Jastrzębska I (2017). Analysis of the Role of CX3CL1 (Fractalkine) and Its Receptor CX3CR1 in Traumatic Brain and Spinal Cord Injury: Insight into Recent Advances in Actions of Neurochemokine Agents. Mol Neurobiol.

[250] Hilla AM, Diekmann H, Fischer D (2017). Microglia Are Irrelevant for Neuronal Degeneration and Axon Regeneration after Acute Injury. J Neurosci.

[251] Fumagalli S, Perego C, Pischiutta F, Zanier ER, De Simoni MG (2015). The ischemic environment drives microglia and macrophage function. Front Neurol.

[252] Gelosa P, Lecca D, Fumagalli M, Wypych D, Pignieri A, Cimino M, Verderio C, Enerbäck M, Nikookhesal E, Tremoli E, Abbracchio MP, Sironi L (2014). Microglia is a key player in the reduction of stroke damage promoted by the new antithrombotic agent ticagrelor. J Cereb Blood Flow Metab.

[253] Lelekov-Boissard T, Chapuisat G, Boissel JP, Grenier E, Dronne MA (2009). Exploration of beneficial and deleterious effects of inflammation in stroke: dynamics of inflammation cells. Philos Trans A Math Phys Eng Sci.

[254] Tonchev AB, Boneva NB, Kaplamadzhiev DB, Kikuchi M, Mori Y, Sahara S, Yamashima T (2008). Expression of neurotrophin receptors by proliferating glia in postischemic hippocampal CA1 sector of adult monkeys. J Neuroimmunol.

[255] Kim E, Cho S (2016). Microglia and Monocyte-Derived Macrophages in Stroke. Neurotherapeutics.

[256] Strecker JK, Schmidt A, Schäbitz WR, Minnerup J (2017). Neutrophil granulocytes in cerebral ischemia - Evolution from killers to key players. Neurochem Int.

[257] Maysami S, Haley MJ, Gorenkova N, Krishnan S, McColl BW, Lawrence CB (2015). Prolonged diet-induced obesity in mice modifies the inflammatory response and leads to worse outcome after stroke. J Neuroinflammation.

[258] Teo JD, Morris MJ, Jones NM (2017). Maternal obesity increases inflammation and exacerbates damage following neonatal hypoxic-ischaemic brain injury in rats. Brain Behav Immun.

[259] Bickford PC, Flowers A, Grimmig B (2017). Aging leads to altered microglial function that reduces brain resiliency increasing vulnerability to neurodegenerative diseases. Exp Gerontol.

[260] Koellhoffer EC, McCullough LD, Ritzel RM (2017). Old Maids: Aging and Its Impact on Microglia Function. Int J Mol Sci.

[261] Eyo UB, Murugan M, Wu LJ (2017). Microglia-Neuron Communication in Epilepsy. Glia.

[262] Grace PM, Hutchinson MR, Maier SF, Watkins LR (2014). Pathological pain and the neuroimmune interface. Nat Rev Immunol.

[263] Naaldijk YM, Bittencourt MC, Sack U, Ulrich H (2016). Kinins and microglial responses in bipolar disorder: a neuroinflammation hypothesis. Biol Chem.

[264] Yirmiya R, Rimmerman N, Reshef R (2015). Depression as a microglial disease. Trends Neurosci.

[265] Lee JK, Tran T, Tansey MG (2009). Neuroinflammation in Parkinson’s disease. J Neuroimmune Pharmacol.

[266] Furman D, Chang J, Lartigue L, Bolen CR, Haddad F, Gaudilliere B, Ganio EA, Fragiadakis GK, Spitzer MH, Douchet I, Daburon S, Moreau JF, Nolan GP (2017). Expression of specific inflammasome gene modules stratifies older individuals into two extreme clinical and immunological states. Nat Med.

[267] Shaw AC, Goldstein DR, Montgomery RR (2013). Age-dependent dysregulation of innate immunity. Nat Rev Immunol.

[268] Schreuder L, Eggen BJ, Biber K, Schoemaker RG, Laman JD, de Rooij SE (2017). Pathophysiological and behavioral effects of systemic inflammation in aged and diseased rodents with relevance to delirium: A systematic review. Brain Behav Immun.

[269] Solano Fonseca R, Mahesula S, Apple DM, Raghunathan R, Dugan A, Cardona A, O’Connor J, Kokovay E (2016). Neurogenic Niche Microglia Undergo Positional Remodeling and Progressive Activation Contributing to Age-Associated Reductions in Neurogenesis. Stem Cells Dev.

[270] Safaiyan S, Kannaiyan N, Snaidero N, Brioschi S, Biber K, Yona S, Edinger AL, Jung S, Rossner MJ, Simons M (2016). Age-related myelin degradation burdens the clearance function of microglia during aging. Nat Neurosci.

[271] Grabert K, Michoel T, Karavolos MH, Clohisey S, Baillie JK, Stevens MP, Freeman TC, Summers KM, McColl BW (2016). Microglial brain region-dependent diversity and selective regional sensitivities to aging. Nat Neurosci.

[272] Maurya SK, Mishra R (2017). Pax6 interacts with Iba1 and shows age-associated alterations in brain of aging mice. J Chem Neuroanat.

[273] Mosher KI, Wyss-Coray T (2014). Microglial dysfunction in brain aging and Alzheimer’s disease. Biochem Pharmacol.

[274] Raj D, Yin Z, Breur M, Doorduin J, Holtman IR, Olah M, Mantingh-Otter IJ, Van Dam D, De Deyn PP, den Dunnen W, Eggen BJ, Amor S, Boddeke E (2017). Increased White Matter Inflammation in Aging- and Alzheimer’s Disease Brain. Front Mol Neurosci.

[275] Bhatt RS, Kothari ST, Gohil DJ, D’Souza M, Chowdhary AS (2015). Novel evidence of microglial immune response in impairment of Dengue infection of CNS. Immunobiology.

[276] Jhan MK, Tsai TT, Chen CL, Tsai CC, Cheng YL, Lee YC, Ko CY, Lin YS, Chang CP, Lin LT, Lin CF (2017). Dengue virus infection increases microglial cell migration. Sci Rep.

[277] Pflugrad H, Meyer GJ, Dirks M, Raab P, Tryc AB, Goldbecker A, Worthmann H, Wilke F, Boellaard R, Yaqub M, Berding G, Weissenborn K (2016). Cerebral microglia activation in hepatitis C virus infection correlates to cognitive dysfunction. J Viral Hepat.

[278] Lannes N, Neuhaus V, Scolari B, Kharoubi-Hess S, Walch M, Summerfield A, Filgueira L (2017). Interactions of human microglia cells with Japanese encephalitis virus. Virol J.

[279] Lannes N, Summerfield A, Filgueira L (2017). Regulation of inflammation in Japanese encephalitis. J Neuroinflammation.

[280] Lum FM, Low DK, Fan Y, Tan JJ, Lee B, Chan JK, Rénia L, Ginhoux F, Ng LF (2017). Zika Virus Infects Human Fetal Brain Microglia and Induces Inflammation. Clin Infect Dis.

[281] Meertens L, Labeau A, Dejarnac O, Cipriani S, Sinigaglia L, Bonnet-Madin L, Le Charpentier T, Hafirassou ML, Zamborlini A, Cao-Lormeau VM, Coulpier M, Missé D, Jouvenet N (2017). Axl Mediates ZIKA Virus Entry in Human Glial Cells and Modulates Innate Immune Responses. Cell Reports.

[282] Shao Q, Herrlinger S, Yang SL, Lai F, Moore JM, Brindley MA, Chen JF (2016). Zika virus infection disrupts neurovascular development and results in postnatal microcephaly with brain damage. Development.

[283] Gnann JW, Whitley RJ (2017). Herpes Simplex Encephalitis: an Update. Curr Infect Dis Rep.

[284] Carpenter JE, Clayton AC, Halling KC, Bonthius DJ, Buckingham EM, Jackson W, Dotzler SM, Card JP, Enquist LW, Grose C (2015). Defensive Perimeter in the Central Nervous System: Predominance of Astrocytes and Astrogliosis during Recovery from Varicella-Zoster Virus Encephalitis. J Virol.

[285] Tso FY, Sawyer A, Kwon EH, Mudenda V, Langford D, Zhou Y, West J, Wood C (2017). Kaposi’s Sarcoma-Associated Herpesvirus Infection of Neurons in HIV-Positive Patients. J Infect Dis.

[286] Tauber SC, Staszewski O, Prinz M, Weis J, Nolte K, Bunkowski S, Brück W, Nau R (2016). HIV encephalopathy: glial activation and hippocampal neuronal apoptosis, but limited neural repair. HIV Med.

[287] Yadav A, Collman RG (2009). CNS inflammation and macrophage/microglial biology associated with HIV-1 infection. J Neuroimmune Pharmacol.

[288] Guo YJ, Luo T, Wu F, Mei YW, Peng J, Liu H, Li HR, Zhang SL, Dong JH, Fang Y, Zhao L (2015). Involvement of TLR2 and TLR9 in the anti-inflammatory effects of chlorogenic acid in HSV-1-infected microglia. Life Sci.

[289] Rao VR, Ruiz AP, Prasad VR (2014). Viral and cellular factors underlying neuropathogenesis in HIV associated neurocognitive disorders (HAND). AIDS Res Ther.

[290] Li F, Wang Y, Yu L, Cao S, Wang K, Yuan J, Wang C, Wang K, Cui M, Fu ZF (2015). Viral Infection of the Central Nervous System and Neuroinflammation Precede Blood-Brain Barrier Disruption during Japanese Encephalitis Virus Infection. J Virol.

[291] Wu X, Liu L, Cheung KW, Wang H, Lu X, Cheung AK, Liu W, Huang X, Li Y, Chen ZW, Chen SM, Zhang T, Wu H, Chen Z (2016). Brain Invasion by CD4(+) T Cells Infected with a Transmitted/Founder HIV-1BJZS7 During Acute Stage in Humanized Mice. J Neuroimmune Pharmacol.

[292] Miner JJ, Diamond MS (2016). Mechanisms of restriction of viral neuroinvasion at the blood-brain barrier. Curr Opin Immunol.

[293] Yun SI, Lee YM (2017). Zika virus: an emerging flavivirus. J Microbiol.

[294] Abou-El-Ardat K, Seifert M, Becker K, Eisenreich S, Lehmann M, Hackmann K, Rump A, Meijer G, Carvalho B, Temme A, Schackert G, Schröck E, Krex D, Klink B (2017). Comprehensive molecular characterization of multifocal glioblastoma proves its monoclonal origin and reveals novel insights into clonal evolution and heterogeneity of glioblastomas. Neuro-oncol.

[295] Grauwet K, Chiocca EA (2016). Glioma and microglia, a double entendre. Nat Immunol.

[296] Gu R, Zhang X, Zhang G, Tao T, Yu H, Liu L, Dou Y, Li A, Qin J (2017). Probing the Bi-directional Interaction Between Microglia and Gliomas in a Tumor Microenvironment on a Microdevice. Neurochem Res.

[297] Miyauchi JT, Tsirka SE (2016). Defining differential roles for microglia and infiltrating macrophages in the growth and neovascularization of glioma. Transl Cancer Res.

[298] Bayerl SH, Niesner R, Cseresnyes Z, Radbruch H, Pohlan J, Brandenburg S, Czabanka MA, Vajkoczy P (2016). Time lapse *in vivo* microscopy reveals distinct dynamics of microglia-tumor environment interactions-a new role for the tumor perivascular space as highway for trafficking microglia. Glia.

[299] Chang AL, Miska J, Wainwright DA, Dey M, Rivetta CV, Yu D, Kanojia D, Pituch KC, Qiao J, Pytel P, Han Y, Wu M, Zhang L (2016). CCL2 Produced by the Glioma Microenvironment Is Essential for the Recruitment of Regulatory T Cells and Myeloid-Derived Suppressor Cells. Cancer Res.

[300] Gieryng A, Kaminska B (2016). Myeloid-derived suppressor cells in gliomas. Contemp Oncol (Pozn).

[301] Ellert-Miklaszewska A, Wisniewski P, Kijewska M, Gajdanowicz P, Pszczolkowska D, Przanowski P, Dabrowski M, Maleszewska M, Kaminska B (2016). Tumour-processed osteopontin and lactadherin drive the protumorigenic reprogramming of microglia and glioma progression. Oncogene.

[302] Hwang JS, Jung EH, Kwon MY, Han IO (2016). Glioma-secreted soluble factors stimulate microglial activation: the role of interleukin-1β and tumor necrosis factor-α. J Neuroimmunol.

[303] Zhang H, Zhang W, Sun X, Dang R, Zhou R, Bai H, Ben J, Zhu X, Zhang Y, Yang Q, Xu Y, Chen Q (2016). Class A1 scavenger receptor modulates glioma progression by regulating M2-like tumor-associated macrophage polarization. Oncotarget.

[304] Dello Russo C, Lisi L, Tentori L, Navarra P, Graziani G, Combs CK (2017). Exploiting Microglial Functions for the Treatment of Glioblastoma. Curr Cancer Drug Targets.

[305] Astell KR, Sieger D (2017). Investigating microglia-brain tumor cell interactions *in vivo* in the larval zebrafish brain. Methods Cell Biol.

[306] Chang KC, Shieh B, Petrash JM (2016). Aldose reductase mediates retinal microglia activation. Biochem Biophys Res Commun.

[307] Chhor V, Moretti R, Le Charpentier T, Sigaut S, Lebon S, Schwendimann L, Oré MV, Zuiani C, Milan V, Josserand J, Vontell R, Pansiot J, Degos V (2017). Role of microglia in a mouse model of paediatric traumatic brain injury. Brain Behav Immun.

[308] Santos CC, Araújo FM, Ferreira RS, Silva VB, Silva JH, Grangeiro MS, Soares ÉN, Pereira ÉP, Souza CS, Costa SL, Segura-Aguilar J, Silva VD (2017). Aminochrome induces microglia and astrocyte activation. Toxicol In Vitro.

[309] Moritz C, Berardi F, Abate C, Peri F (2015). Live imaging reveals a new role for the sigma-1 (σ1) receptor in allowing microglia to leave brain injuries. Neurosci Lett.

[310] Garcia-Mesa Y, Jay TR, Checkley MA, Luttge B, Dobrowolski C, Valadkhan S, Landreth GE, Karn J, Alvarez-Carbonell D (2017). Immortalization of primary microglia: a new platform to study HIV regulation in the central nervous system. J Neurovirol.

[311] Mizee MR, Miedema SS, van der Poel M, Adelia, Schuurman KG, van Strien ME, Melief J, Smolders J, Hendrickx DA, Heutinck KM, Hamann J, Huitinga I (2017). Isolation of primary microglia from the human post-mortem brain: effects of ante- and post-mortem variables. Acta Neuropathol Commun.

[312] Olah M, Raj D, Brouwer N, De Haas AH, Eggen BJ, Den Dunnen WF, Biber KP, Boddeke HW (2012). An optimized protocol for the acute isolation of human microglia from autopsy brain samples. Glia.

[313] Napoli I, Kierdorf K, Neumann H (2009). Microglial precursors derived from mouse embryonic stem cells. Glia.

[314] Douvaras P, Sun B, Wang M, Kruglikov I, Lallos G, Zimmer M, Terrenoire C, Zhang B, Gandy S, Schadt E, Freytes DO, Noggle S, Fossati V (2017). Directed Differentiation of Human Pluripotent Stem Cells to Microglia. Stem Cell Reports.

[315] Haenseler W, Sansom SN, Buchrieser J, Newey SE, Moore CS, Nicholls FJ, Chintawar S, Schnell C, Antel JP, Allen ND, Cader MZ, Wade-Martins R, James WS, Cowley SA (2017). A Highly Efficient Human Pluripotent Stem Cell Microglia Model Displays a Neuronal-Co-culture-Specific Expression Profile and Inflammatory Response. Stem Cell Reports.

[316] Takata K, Kozaki T, Lee CZ, Thion MS, Otsuka M, Lim S, Utami KH, Fidan K, Park DS, Malleret B, Chakarov S, See P, Low D (2017). Induced-Pluripotent-Stem-Cell-Derived Primitive Macrophages Provide a Platform for Modeling Tissue-Resident Macrophage Differentiation and Function. Immunity.

[317] Etemad S, Zamin RM, Ruitenberg MJ, Filgueira L (2012). A novel *in vitro* human microglia model: characterization of human monocyte-derived microglia. J Neurosci Methods.

[318] Leone C, Le Pavec G, Même W, Porcheray F, Samah B, Dormont D, Gras G (2006). Characterization of human monocyte-derived microglia-like cells. Glia.

[319] Ohgidani M, Kato TA, Setoyama D, Sagata N, Hashimoto R, Shigenobu K, Yoshida T, Hayakawa K, Shimokawa N, Miura D, Utsumi H, Kanba S (2014). Direct induction of ramified microglia-like cells from human monocytes: dynamic microglial dysfunction in Nasu-Hakola disease. Sci Rep.

[320] Beins E, Ulas T, Ternes S, Neumann H, Schultze JL, Zimmer A (2016). Characterization of inflammatory markers and transcriptome profiles of differentially activated embryonic stem cell-derived microglia. Glia.

[321] Ponomarev ED, Novikova M, Maresz K, Shriver LP, Dittel BN (2005). Development of a culture system that supports adult microglial cell proliferation and maintenance in the resting state. J Immunol Methods.

[322] Spangenberg EE, Green KN (2017). Inflammation in Alzheimer’s disease: lessons learned from microglia-depletion models. Brain Behav Immun.

